# OCT4 interprets and enhances nucleosome flexibility

**DOI:** 10.1093/nar/gkac755

**Published:** 2022-09-22

**Authors:** Caitlin M MacCarthy, Jan Huertas, Claudia Ortmeier, Hermann vom Bruch, Daisylyn Senna Tan, Deike Reinke, Astrid Sander, Tim Bergbrede, Ralf Jauch, Hans R Schöler, Vlad Cojocaru

**Affiliations:** Department of Cellular and Developmental Biology, Max Planck Institute for Molecular Biomedicine, Münster, Germany; Department of Cellular and Developmental Biology, Max Planck Institute for Molecular Biomedicine, Münster, Germany; Yusuf Hamied Department of Chemistry, University of Cambridge, UK; Department of Cellular and Developmental Biology, Max Planck Institute for Molecular Biomedicine, Münster, Germany; Department of Cellular and Developmental Biology, Max Planck Institute for Molecular Biomedicine, Münster, Germany; School of Biomedical Sciences, Li Ka Shing Faculty of Medicine, University of Hong Kong, Hong Kong SAR, China; Max Planck Institute of Molecular Physiology, Dortmund, Germany; Max Planck Institute of Molecular Physiology, Dortmund, Germany; Lead Discovery Center GmbH, Dortmund, Germany; School of Biomedical Sciences, Li Ka Shing Faculty of Medicine, University of Hong Kong, Hong Kong SAR, China; Department of Cellular and Developmental Biology, Max Planck Institute for Molecular Biomedicine, Münster, Germany; Medical Faculty, University of Münster, Germany; Department of Cellular and Developmental Biology, Max Planck Institute for Molecular Biomedicine, Münster, Germany; Computational Structural Biology Group, University of Utrecht, The Netherlands; STAR-UBB Institute, Babeş-Bolyai University, Cluj-Napoca, Romania

## Abstract

Pioneer transcription factors are proteins that induce cellular identity transitions by binding to inaccessible regions of DNA in nuclear chromatin. They contribute to chromatin opening and recruit other factors to regulatory DNA elements. The structural features and dynamics modulating their interaction with nucleosomes are still unresolved. From a combination of experiments and molecular simulations, we reveal here how the pioneer factor and master regulator of pluripotency, Oct4, interprets and enhances nucleosome structural flexibility. The magnitude of Oct4’s impact on nucleosome dynamics depends on the binding site position and the mobility of the unstructured tails of nucleosomal histone proteins. Oct4 uses both its DNA binding domains to propagate and stabilize open nucleosome conformations, one for specific sequence recognition and the other for nonspecific interactions with nearby regions of DNA. Our findings provide a structural basis for the versatility of transcription factors in engaging with nucleosomes and have implications for understanding how pioneer factors induce chromatin dynamics.

## INTRODUCTION

In eukaryotic cells, the DNA containing all the information about the cell’s fate and function is packed into chromatin inside the nucleus. The nucleosome, the basic unit of chromatin, is a nucleoprotein complex formed by 145–147 base pairs of DNA wrapped around an octamer of four histones proteins (H3, H4, H2A and H2B). Histones have a globular domain and disordered, charged regions at the N-terminus (and at the C-terminus of histone H2A), known as the histone tails (1).

The reading and regulation of the genetic information is carried out by site-specific DNA binding proteins called transcription factors (TFs) (2). The extent of genome packing leaves a sizeable fraction of the DNA inaccessible for the majority of TF binding due to occlusion of binding sites by the histone core. Interestingly, a subgroup of TFs known as pioneer TFs (pTFs) can recognize and bind their motifs in nucleosome occupied regions of the genome (3–5). A recent systematic study revealed that several families of transcription factors can have pioneer activity, binding with different affinities and mechanisms to nucleosomes (6).

One particularly interesting pTF is Oct4, a master regulator of pluripotency (7). Oct4 cooperates with pTFs Sox2 and Klf4 in the conversion of somatic cells to pluripotent stem cells during reprogramming (8). Structurally, Oct4 contains a DNA binding domain divided into two subdomains, the POU specific domain (POU_S_) and the POU homeodomain (POU_HD_), connected by a flexible linker. Together, the two subdomains recognize eight sequential base pairs in free DNA (9): ATGC by the POU_S_ and [A/T]AAT by the POU_HD_. On nucleosomes Oct4 recognizes only half of its eight base pair binding site due to the twist of DNA and steric clashes with the histone core, which inhibit the canonical binding of the second subdomain (10–14).

Oct4 is known to bind cooperatively with Sox2 in the genome (15–22). Recent studies suggest they may also work together as pioneers on nucleosomal DNA (10,11,23), where one TF binding event alters DNA-histone contacts and DNA positioning, facilitating the binding of the second TF (13,14,22,24). However, the molecular mechanisms of pTF binding to the nucleosome and higher order chromatin structures, the effect pTF binding has on chromatin dynamics at different levels, and the role of pTFs binding in propagating the chromatin rearrangements needed for cellular transitions are not understood.

The first structure of a pTF-nucleosome complex resolved experimentally suggested a mechanism by which Sox2 binds and unravels nucleosomes (25). Following up on this, another structure revealed Oct4 together with Sox2 bound to a nucleosome (13). However, both structures are missing essential parts. In the first, the entire outer gyres of the nucleosomal DNA were not resolved, whereas in the second only the Oct4 POU_S_ was resolved. The histone tails were not resolved in either. These and many other structural studies of nucleosome interactions use synthetic DNA sequences, which bind tightly to the histone core. These are well-suited for structural techniques, but have limited relevance for nucleosome behavior *in vivo*.

Echigoya *et al.* resolved the structure of a genomic nucleosome that can be bound by Oct4 (12,26). While they did not present the structure of the Oct4-nucleosome complex, they revealed a novel Oct4 binding site, different from those previously reported by Soufi *et al.* (11). Echigoya *et al.* speculated that the POU_S_ subdomain of Oct4 recognizes this binding site (12). Moreover, they showed that Oct4 interacts with histone H3 and competes with the linker histone for binding. Both this study and the work of Michael *et al.* (13) highlighted the importance of the POU_S_ but not the POU_HD_ subdomain in Oct4’s nucleosome binding. Nevertheless, molecular simulations using a reduced (coarse-grained) representation of the molecular species revealed binding of Oct4 to this novel site (14), but with the POU_HD_ domain recognizing the site. This is also supported by the DNA sequence that bares a typical homeodomain binding site.

We previously used molecular modeling and short molecular dynamics (MD) simulations to build models of Oct4 bound to nucleosomes on the binding sites proposed by Soufi *et al.* (10,11). From these, we confirmed that the canonical configuration of Oct4 with the subdomains bound on opposite faces of DNA is incompatible with nucleosome binding and we proposed alternative binding modes (27).

Here we report atomic resolution structural features and the dynamics involved in the Oct4 interaction with two genomic nucleosomes that differ in the number and positions of the Oct4 binding sites. We first validated the sequence specific binding of Oct4 to both nucleosomes and probed how this is affected by nucleosome flexibility using *in vitro* biochemical experiments. Then, we turned to atomistic MD simulations to understand how Oct4 binds to its motifs and how it modifies the structural flexibility of the nucleosomes. The two nucleosomes have sequences from regulatory elements of LIN28B and the ESRRB genes. We selected these from the earliest data set demonstrating Oct4’s pioneer function (10) for their importance in defining stem cell pluripotency (28–31), their activation after 48 h of pluripotency induction (RNA-seq), and their distinct arrangement of Oct4 binding sites. We found that Oct4 favors binding sites positioned near the DNA ends, recognizes its binding using either DNA binding subdomain, and requires some nucleosome structural flexibility for efficient binding. Oct4 also modifies nucleosome breathing and stabilizes open nucleosome conformations depending on the location of the binding site, the mobility of histone tails, and the motions of its DNA binding sudomains.

## MATERIALS AND METHODS

### Full-length Oct4 expression and purification

Experiments were all performed with purified full-length Oct4 from *Mus musculus*. Briefly, Oct4 was cloned into the pOPIN expression vector using the SLIC method and Phusion Flash High-Fidelity PCR Master Mix (Finnzymes/New England Biolabs). SLIC reactions were then transformed into One Shot™ OmniMAC™ 2 T1^®^ Chemically Competent *Escherichia coli* (ThermoFisher Scientific; Waltham, MA). After sequencing, the pOPIN-cHis-Oct4 construct was co-transfected with flashBACULTRA™ bacmid DNA (Oxford Expression Technologies; Oxford, UK) into Sf9 cells (ThermoFisher Scientific) using Cellfectin II^®^ (ThermoFisher Scientific) to generate recombinant baculovirus. Mid-log phase Sf9 cells were used to amplify the virus. Suspension High Five™ cells were infected with P3 virus for two days at 27°C and 120 rpm shaking. After expression, crude lysates were purified on a HiTrap TALON column (GE Healthcare; Chicago, IL), cleaved on the column with 3C protease and followed by size exclusion chromatography (HiLoad Superdex 200, GE Healthcare). The final product was collected in 25 mM HEPES pH 7.8, 150 mM NaCl, 1 mM TCEP, and 5% glycerol with around 95% purity confirmed by SDS-PAGE.

### Nucleosome reconstitution

The LIN28B and ESRRB nucleosome sequences were taken from Soufi *et al.* (10) (Hg18 chr6:105, 638,004-105,638,165 and Hg18 chr14:75,995,474-75,995,636, respectively, see also Supplementary Methods). WT and mutant sequences were purchased from IDT (Coralville, IA, USA) with flanking AvaI restriction sites, sequenced, amplified in *E. coli*, digested, and finally purified by native PAGE using electroelution. After Cy5 labeling, DNAs were reconstituted at DNA:octamer ratios ranging from 1:1.2 to 1:1.6 with purified full-length *Drosophila melanogaster* histone octamer using the salt-gradient dialysis method previously described (32), final buffer composition: 10 mM HEPES pH 7.6, 50 mM NaCl, 1 mM EDTA, and 0.5 mM DTT. Following dialysis, nucleosomes were heat shifted at 37°C for 2 h and then checked for quality and concentration by native PAGE. Histone stoichiometry was checked by $22 \%$ SDS-PAGE followed by coomassie (R-250) (SERVA, Heidelberg, Germany) staining.

### Electrophoretic mobility shifts and competition assays

For binding reactions, 20 nM nucleosomes were incubated with 0.05–0.4 μM of purified Oct4 in binding buffer (25 mM HEPES pH 7.6, 50 mM NaCl, 0.5 mM EDTA, 1 μg/μl BSA, 0.8 mM DTT, and 10% glycerol) for 1 h at 25°C. After incubation, reactions were run directly on a 6% native polyacrylamide gel (acrylamide/bis-, 37.5/1) containing 27 mM Tris-borate, 0.6 mM EDTA, and 5% glycerol and run in the same buffer. All Cy5-labeled DNA was detected using Fujifilm FLA-9000 (GE Healthcare). Competition assays using specific and nonspecific oligos were performed as previously described (11) using 0.2-4 μM of competitor, 20 nM nucleosome, and 105 nM Oct4. Oct4 dissociation from nucleosomes was determined by incubating specific competitor with the pre-formed Oct4/nucleosome complex that occurs after 1 h incubation at 25°C. Off-rate conditions were empirically determined for LIN28B and ESRRB nucleosomes due to the substantial differences in complex stability: LIN28B – 9 nM nucleosome, 45 nM Oct4 and 0.2-3.5 μM unlabeled competitor at 25°C for 30 min; and ESRRB – 9 nM nucleosome, 67.5 nM Oct4, and 9–90 nM unlabeled competitor at 25°C for 5 min. A short oligo containing the ESRRB Oct4 binding sequence was used as competitor for the ESRRB nucleosomes: AAGTGATAGTTATGCAGAGCGAATGGAGGG. For LIN28B, the specific competitor sequence published in Soufi *et al.* was used (11). Experiments were performed in triplicate and densitometry was carried out using a DNA standard curve and Quantity One^®^ software (Bio-Rad, Hercules, CA). The values reported in Figure 2 were calculated by dividing the portion of Oct4-nucleosome complex by the value of starting free nucleosome (control). Two-way ANOVA using Sidak’s multiple comparison test was performed on relevant mean and standard deviation values. Statistics were done using Prism 7.0a for Mac. We also estimated the apparent dissociation constants of Oct4 from nucleosomes (see Supplementary Methods).

### Crosslinking experiments

Assemblies were generated as described in a previous section. Half of the assembly preparation was incubated with a final concentration of 1% formaldehyde on ice for 15 min. Crosslinking reactions were quenched by adding glycine to 250 mM final concentration. Crosslinking efficiency was checked by incubating an aliquot of crosslinked and uncrosslinked nucleosome in 15 mM MgCl_2_ and 300 mM NaCl at 60^○^C for 15 min and then running the samples on a 6% native PA gel. Samples were purified on a 10–30% sucrose gradient spun at 30 000 × g and 4^○^C in a Beckman Coulter Optima L-100 XP swing bucket rotor (SW-41; Brea, CA) for 18 h. Fractions were collected from the bottom of the gradient, screened on native gels, pooled, and quantitated by densitometry using a DNA standard curve and Quantity One^®^ software (Bio-Rad, Hercules, CA). Off-rates with crosslinked nucleosomes were performed as described in the previous section.

### Modelling Oct4-nucleosome complexes

To build the structural models of the LIN28B and ESRRB nucleosomes, we first selected 168 base pair sequences from the human genome from the data by Soufi *et al.* (10,11). The selection was based on a comparison of ChIP-Seq data (accession code GSE36570) for Oct4 binding during reprogramming of fibroblasts to pluripotency (48 h after induction of Oct4 and the other three transcription factors required) with MNase-seq data revealing nucleosome positioning in human fibroblasts (accession code GSM543311) (33). We considered the region around the MNase peak to correspond to the dyad and optimized the accessibility of the Oct4 binding sites proposed by Soufi *et al.* (S^–1.5^ and HD^–4.5^ on LIN28B and S^+5.5^ on ESRRB) (10,11) (see Supplementary Methods). Then we threaded these selected sequences on a 168 base pairs nucleosome with the original Widom 601 DNA sequence (34) and with *Drosophila* or human histones by swapping each base to the new sequence with the ‘swapna’ function in Chimera (35). The complete 168 base pair Widom nucleosomes were built using the DNA from the 3LZ0 structure with the histones (including the histone tails) modelled using the 2PYO and 1KX5 structures as templates (see Supplementary Methods). We previously performed extensive MD simulations with these nucleosomes (36).

Next, we modelled Oct4-nucleosome complexes using the LIN28B and ESRRB DNA sequences from the human genome with *Drosophila melanogaster* and human histones for the LIN28B and ESRRB nucleosomes respectively (36) (see Supplementary Methods for details). The initial configuration of Oct4 we took from the following structures: (i) Oct4 bound to DNA in the canonical configuration with the two subdomains bound on opposite sides of DNA (37) that were built based on the structure of Oct4 bound as a homodimer to the PORE motif (38). (ii) Oct4 bound with both subdomains on the same side of DNA (MORE configuration) obtained from the structure of Oct4 bound as the homodimer to the MORE motif (39) by stripping one monomer. (iii) Oct4 configurations obtained from a 100 ns MD simulation of apo Oct4 (27) (see MD protocol below).

To build models of Oct4-nucleosome complexes, we superposed the binding sites from the structures of Oct4 bound to free DNA to the binding sites on the nucleosomes and removed the free DNA. We superposed the MD-generated configurations of Oct4 on the initial models of canonical Oct4-nucleosome complexes (27) by fitting the subdomain that binds specifically to the DNA and removed the canonical configuration of Oct4. The resulting models of Oct4-nucleosome complexes had no steric clashes between the nonspecifically bound subdomain and the core histones. All models were validated in initial 100 ns classical MD simulations (protocol below). The short simulations validating the complexes of Oct4 bound to the LIN28B nucleosome on the binding sites proposed by Soufi *et al.* (HD^–4.5^ and S^–1.5^) were presented in our previous study (27).

The models of free and Oct4-bound tail-less nucleosomes were obtained by removing all histone tails from the corresponding nucleosome and Oct4-nucleosome models after equilibration. The histone tails were defined as follows: residues 1–45 for H3 (Human and Drosophila), 1–32 for H4 (human and Drosophila), 1–18 and 119–129 for human H2A, 1–17 and 116–124 for Drosophila H2A, 1–33 for Human H2B and 1–31 for Drosophila H2B. 146 DNA basepairs centered on the dyad were defined as the nucleosome core DNA, whereas the remaining 11 basepair on each side were defined as the linker DNAs (L-DNAs). The outer gyres were defined as the last 40 bp at each end. The central 88 basepairs were considered as the inner gyre.

### Molecular dynamics simulations

Classical MD (cMD) simulations were performed as previously described (27,36). Every simulated species was first solvated in a truncated octahedron box of SPC/E water molecules, with a layer of at least 12-15 Å of water around the solute. All systems had ∼350 000 atoms. Na^+^ ions were added to counter the negative charges of the system. K^+^ and Cl^–^ ions were added, up to a concentration of 150 mM. The systems were then optimized with an energy minimization, performed with the AMBER software (40). Then, the systems were equilibrated for 13.5 ns, using *NAMD* (41). The equilibration protocol was adapted from Jerabek *et al.* (39) and is described in detail in the Supplementary Methods. Harmonic distance restraints were applied to maintain DNA base pairing and Oct4–DNA base interactions. The force constant for these was gradually decreased. At the latest stages, the equilibration was unrestrained. Then, we performed production simulations in *NAMD*, in the isobaric-isothermic (NPT, *p* = 1 atm, *T* = 300 K) ensemble, with Langevin dynamics for temperature control and a Nosé-Hoover and Langevin piston for pressure control. The Li-Merz ion parameters (42), the ff14SB (43) and the parmbsc1 force fields (44) were used for ions, protein, and DNA, respectively. Each individual simulation was 1 or 2 μs long and multiple replicas were performed (Table 1).

**Table 1.** Overview of the simulations performed

						Number of Oct4–DNA contacts^c^			
Simulation name^a^	DNA	Oct4 site	Starting structure^b^	Time	R_g_^c^	POU_S_-Bases	POU_S_-Backbone	POU_HD_-Bases	POU_HD_-Backbone
LIN28B_1_	LIN28B	–	Free nuc	1 μs	49.2 (48.3–51.0)	–	–	–	–
LIN28B_2_	LIN28B	–	Free nuc	1 μs	48.7 (48.0–49,9)	–	–	–	–
ESRRB_1_	ESRRB	–	Free nuc	1 μs	48.6 (47.8–51.3)	–	–	–	–
ESRRB_1–b_	ESRRB	–	1 μs of ESRRB_1_	1 μs	50.2 (48.8–52.5)	–	–	–	–
ESRRB_2_	ESRRB	–	Free nuc	1 μs	47.6 (47.1–48.2)	–	–	–	–
HD^–7^rev_1_	LIN28B	HD^–7^	Cano Oct4	1 μs	49.8 (49.3–50.4)	14 (5–28)	77 (26–99)	107 (86–126)	208 (172–250)
HD^–7^rev_2_	LIN28B	HD^–7^	MD Oct4	1 μs	50.1 (49.6–50.6)	3 (0–10)	30 (0–64)	64 (36–81)	133 (96–176)
HD^–7^rev_3_	LIN28B	HD^–7^	Cano Oct4	1 μs	50.3 (49.6–50.9)	95 (33–109)	131 (63–155)	105 (83–131)	214 (147–254)
HD^–7^rev_4_	LIN28B	HD^–7^	MD Oct4	1 μs	49.2 (48.6–49.8)	7 (2–13)	54 (22–98)	62 (40–87)	175 (135–214)
S^+5.5^_1_	ESRRB	S^+5.5^	MD1 Oct4	3 μs	54.5 (52.5–56.3)	118 (102–135)	202 (139–243)	23 (9–39)	135 (67–185)
S^+5.5^_1_ – Oct4	ESRRB	–	1 μs of S^+5.5^_1_	2 μs	58.0 (55.6–59.0)	–	–	–	–
S^+5.5^_2_	ESRRB	S^+5.5^	MD2 Oct4	3 μs	49.5 (48.6–50.0)	94 (75–115)	104 (78–146)	48 (22–71)	59 (27–91)
S^+5.5^_2–b_	ESRRB	S^+5.5^	1 μs of S^+5.5^_2_	1 μs	54.5 (51.3–57.6)	94 (80–108)	163 (136–187)	59 (36–91)	49 (22–78)
S^+5.5^_2–b_ – Oct4	ESRRB	S^+5.5^	1 μs of S^+5.5^_2_	1 μs	51.1 (50.1–53.0)	–	–	–	–
S^+5.5^_3_	ESRRB	S^+5.5^	MD1 Oct4	2 μs	48.2 (47.4–48.9)	123 (99–148)	154 (93–191)	25 (7–35)	95 (62–132
S^+5.5^_4_	ESRRB	S^+5.5^	MD2 Oct4	1 μs	48.7 (48.2–49.3)	74 (55–112)	138 (105–170)	53 (3–87)	83 (40–112)
S^+5.5^_5_	ESRRB	S^+5.5^	MORE Oct4	1 μs	48.2 (47.6–48.9)	97 (82–111)	115 (81–146)	51 (12–64)	157 (57–190)
S^+5.5^_T1_	ESRRB	S^+5.5^	MD1 Oct4	2 μs	52.3 (50.0–56.8)	117 (77–142)	147 (105–187)	10 (0–24)	54 (27–77)
S^+5.5^_T1_ – Oct4	ESRRB	–	1 μs of S^+5.5^_T1_	1 μs	48.0 (47.5–48.7)	–	–	–	–
S^+5.5^_T1–b1_	ESRRB	S^+5.5^	800 ns of S^+5.5^_T1_	250 ns	56.1 (54.9–58.2)	98 (73–123)	146 (112–183)	1 (0–15)	30 (0–71)
S^+5.5^_T2_	ESRRB	S^+5.5^	250 ns of S^+5.5^_T1–b1_	1 μs	53.8 (52.6–55.1)	90 (74–104)	139 (104–161)	60 (28–84)	144 (108–177)
S^+5.5^_T1–b2_	ESRRB	S^+5.5^	800 ns of S^+5.5^_T1_	250 ns	55.8 (54.2–58.3)	100 (81–127)	137 (103–169)	3 (0–18)	34 (0–76)
S^+5.5^_T3_	ESRRB	S^+5.5^	200 ns of S^+5.5^_T1–b2_	1 μs	54.1 (53.2–55.6)	111 (85–130)	172 (125–208)	13 (4–56)	137 (84–180)
ESRRB_1_–TL	ESRRB	–	eq of ESRRB_1_	2 μs	51.3 (48.9–57.9)	–	–	–	–
ESRRB_2_–TL	ESRRB	–	eq of ESRRB_2_	2 μs	52.3 (49.3–55.5)	–	–	–	–
S^+5.5^_1_–TL	ESRRB	S^+5.5^	eq of S^+5.5^_1_	2 μs	60.5 (53.9–62.8)	94 (90–112)	155 (111–186)	58 (21–109)	118 (79–154)
S^+5.5^_2_–TL	ESRRB	S^+5.5^	eq of S^+5.5^_2_	2 μs	57.7 (52.4–62.5)	99 (81–125)	159 (122–185)	57 (29–88)	100 (50–129)
S^+5.5^_3_–TL	ESRRB	S^+5.5^	eq of S^+5.5^_3_	2 μs	57.0 (51.1–61.6)	93 (75–111)	122 (89–158)	85 (43–117)	113 (67–151)
S^+5.5^_4_–TL	ESRRB	S^+5.5^	eq of S^+5.5^_4_	2 μs	58.5 (51.9–61.9)	92 (77–107)	135 (97–177)	101 (53–134)	99 (58–133)

^a^ ‘T’ = simulations started with H3 and H2AC tail configurations taken from the open nucleosome conformation established in S^+5.5^_1_; ‘b’ = biased simulations; ‘TL’ = simulations with tail-less nucleosome.

^b^ ‘Cano’, ‘MD’ = Canonical and MD-generated Oct4 configurations; ‘nuc’ = nucleosome; ‘eq’ = equilibration.

^c^ R_g_ and the number of contacts are shown as the median with the percentiles 5 to 95 in brackets; a contact was defined as a non-hydrogen atom closer than 4.5 Å to another non-hydrogen atom.

### Biased molecular dynamics simulations

We used minimal inter-atomic distances and coordination numbers (δ_min_ and *C*) as collective variables for bias MD (bMD) simulations. These are defined in the *Colvar* module (45) implemented in *NAMD* and *VMD* (46) (https://colvars.github.io/). δ_min_ between two groups of atoms were measured using the weighted mean distance collective variable *distanceInv* defined as follows:

$$\begin{equation*}d_{1,2}^{[n]} = \left(\frac{1}{N_1N_2}\sum _{i,j}\left(\frac{1}{||d^{ij}||}\right)^n\right)^{-1/n}\end{equation*}$$

where ||*d*^*ij*^|| is the distance between atoms *i* and *j* in groups 1 and 2 respectively, and *n* is an even integer. This distance will asymptotically approach the minimal distance when increasing *n*. We used *n*=100 because in test calculations we found it approximates best the minimal distance without compromising the measurement of the collective variable. *C* between two groups of atoms were measured as:

$$\begin{equation*}C(group1,group2) = \sum _{i{\epsilon }group1} \sum _{i{\epsilon }group2} \frac{1-(|x_i-x_j|/d_0)^n}{1-(|x_i-x_j|/d_0)^m}\end{equation*}$$

where *x*_*i*_, *x*_*j*_ are the atomic coordinates of atoms *i* and *j*, *d*_0_ is the threshold distance (4.0 Å), *n* and *m* are exponents that control the properties of the function (we used *n* = 6, *m* = 12).

The atom groups were defined using the Cα and P atoms from the proteins and DNA, respectively. To sample nucleosome conformations in which the histone tails do not interact with the linker DNA, we applied harmonic wall biases to keep δ_min_ larger than 12 Å and *C* equal to 0 between the H3 and H2AC tails and the outer DNA gyre.

To sample the motion of the POU_HD_ between DNA gyres, we applied additional steered MD harmonic biases in which δ_min_ between POU_HD_ and the inner and outer gyres were changed with constant velocity from 5 to 15 Å and from 32 to 12 Å ,respectively. To avoid the collapse of the POU_HD_ on the outer gyre but near to the inner gyre, we also applied steered MD to bias the δ_min_ between POU_HD_ and the 3’ L-DNA between 58 and 28 Å. The steered MD biases were applied over 250 ns of simulation. To prevent the rapid closing of the nucleosome before the POU_HD_ moved between the gyres, near the L-DNA, we also applied the harmonic wall biases to keep δ_min_ and *C* between the H3 and H2AC tails and the outer DNA gyre larger than 30 Å and equal to 0, respectively, and to keep the δ_min_ between the two gyres larger than 20 Å.

For all harmonic biases we used a force constant of 10 kcal/mol*Å^2^. After each biased simulation we performed additional, 1 μs long classical simulations with or without Oct4 to ensure that the application of the biases did not distort dynamics.

### Analysis of the MD simulations

We fitted all simulations to the core region of the histones (excluding histone tails) using the initial minimized structure as a reference.

We characterized the breathing motions using the coordinate system originally described by Öztürk *et al.* (47), also used in our previous work (27,36). First, a coordinate system XYZ was defined, with the origin on the dyad. X was described as the vector along the dyad axis, Y as the cross product between X and a vector perpendicular to X intersecting it approximately at the center of the nucleosome, and Z as the cross product between X and Y. Two vectors, v_3_ and v_5_ were defined along the 3’ and 5’ L-DNAs. Then, the angle γ_1_ was defined as the angle between the projection of these vectors in the XZ plane and the Z axis, and γ_2_, as the angle between the projection of the vectors on the XY plane and the Y axis.

The mass-weighted radius of gyration of the DNA (*R*_g_) and the number of contacts between histone tails or Oct4 and the DNA were calculated with *cpptraj* (48). A contact was defined when two non-hydrogen atoms were closer than 4.5 Å. Contacts were considered stable if they were present for more than 75% of the simulation.

## RESULTS

### Oct4 binds to specific sites on nucleosomes using either DNA binding subdomain

**Figure 1.**
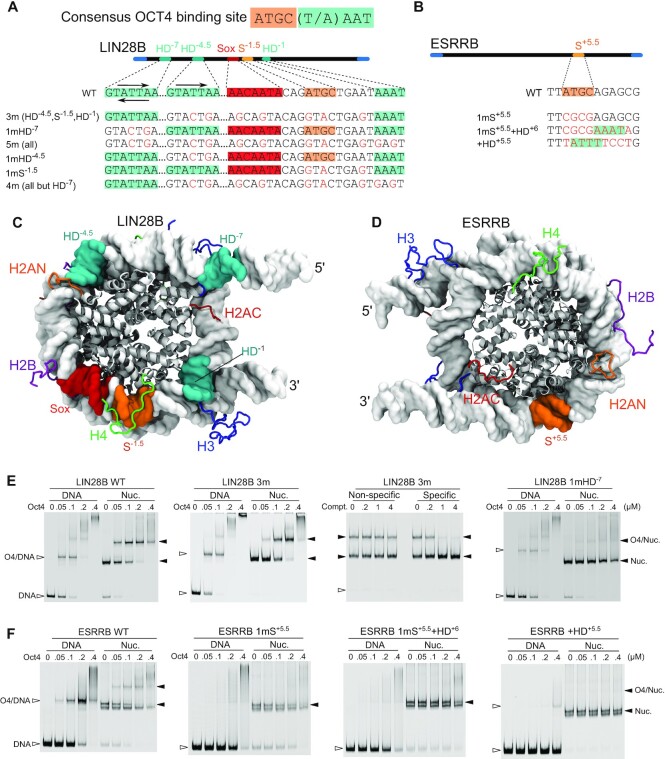
Oct4 binds to native nucleosomes at different positions with either DNA binding subdomain. (A, B) Schematics of the consensus OCT4 binding site, LIN28B (**A**) and ESRRB (**B**) genomic nucleosomes and mutants showing the positioning and sequences of the Oct4 binding sites. The mutants are named with a ‘m’ preceded by the number of the binding sites mutated and followed by the names of these sites. The mutated sites are shown in brackets if there are more than three. The POU_S_, POU_HD_, and Sox2 binding sites are in orange, teal, and red, respectively. Arrows indicate the binding orientation. The POU_S_ binds in a 5’-ATGC-3’ orientation to free DNA, whereas the POU_HD_ in a 5’-T(A)AAT-3’ orientation with the N-terminal tail recognizing the first half and the globular part the second half. (C, D) Structural views of LIN28B (**C**) and ESRRB (**D**) with the histone core in gray cartoons, DNA in white surface, binding sites in the corresponding colors, and histone tails in blue (H3), green (H4), orange (H2AN), red (H2AC), and purple (H2B). These representations and coloring are kept throughout the manuscript. (**E**, **F**) EMSAs of purified Oct4 with free DNA (left) and reconstituted nucleosomes (right). The third gel in 1E shows a competition assay using excess specific and nonspecific competitor. Filled horizontal arrowheads indicate nucleosomes or nucleosome-protein complexes and empty arrowheads, free DNA or DNA–protein complexes.

We first asked how each subdomain of Oct4 contributes to Oct4 binding on the LIN28B and ESRRB nucleosomes. To address this, we characterized Oct4 binding to a series of native (WT) and mutated nucleosomal DNAs (Figure 1, Supplementary Figures S1, S2A–D). The native sequences we selected depict the diversity of Oct4 genomic binding: one sequence, LIN28B (11), contains multiple sites for Oct4 (Figure 1A) and other pTFs, whereas the other, ESRRB (Figure 1B) has a single Oct4 binding site. On LIN28B, the binding sites are located either towards the 5’ side (using the genomic 5’–3’ orientation as reference) or in the core of the nucleosome (Figure 1C), whereas on ESRRB the binding site lies in the core, closer to the 3’ end (Figure 1D).

We first confirmed Oct4 binding to putative nucleosomal sites by mutating key DNA bases within these sites (Figure 1A, B), and then evaluated nucleosome binding in electrophoretic mobility shift assays (EMSAs). Cy-5 labeled DNAs were assembled into nucleosomes using salt gradient dialysis and the histone core positioning was checked to ensure it was comparable between sequences (Supplementary Figure S1). Mutation of the Oct4 sites and the Sox site proposed by Soufi *et al.* (‘3m’) (11) did not prevent Oct4 binding to LIN28B (Figure 1E). Given that Oct4 is also known to interact with histones (12,49) or nonspecifically with DNA, we added a molar excess of specific or nonspecific unlabeled double-stranded DNA and confirmed that the persistent binding of Oct4 to the 3m mutant nucleosome was sequence specific (Figure 1E). This opened the possibility that additional Oct4 binding sites exist on this nucleosome. A search of the LIN28B sequence revealed two potential homeodomain binding sites at superhelical locations (SHL) –7 and –1 (Figure 1A, C, HD^–7^ and HD^–1^). When we mutated all four Oct4 sites and the Sox site (5m), Oct4 nucleosome binding was reduced substantially (Supplementary Figure S2A,B).

In order to tease out the relative contributions of each site to overall Oct4–LIN28B binding, we mutated each site individually and in combinations leaving one site untouched. We observed that each site contributes to Oct4’s affinity, except for the Sox and HD^-1^ sites (Figure 1E, Supplementary Figure S2C,D). Interestingly, mutating just the HD^–7^ site, 1mHD-7, resulted in loss of Oct4 binding, suggesting that HD^–7^ is the primary Oct4 binding site (Figure 1E). While we performed the experiments and prepared the pre-print of this manuscript, two independent studies reported Oct4 binding to the HD^-7^ site, confirming our findings (12,14). Our results also indicate that the sites previously proposed by Soufi *et al.* (10,11) HD^–4.5^ and S^–1.5^ serve as secondary binding sites.

The HD^–7^ site contains a generic homeodomain binding site preceded by an adjacent POU_S_-like half-site (ATGA, not the canonical ATGC). Nevertheless, mutation of only the HD half-site reduced Oct4 binding substantially, demonstrating that the binding of Oct4 to this site is driven by the POU_HD_ (Figure 1E, 1mHD^-7^), in agreement with the coarse grained simulations by Tan *et al.* (14) but in contrast with Echigoya *et al.* who suggested that the binding is driven by the POU_S_ (12). Moreover, the sequence of the HD–^-7^ and HD^–4.5^ are identical (Figure 1A), indicating Oct4’s preference for the HD^–7^ position and not the DNA sequence alone. This is also reflected in the apparent Oct4 dissociation constants estimated from the EMSAs, where the value for the 1mHD^–7^ nucleosome is approximately four times larger than that for 1mHD^–4.5^ and 12 times WT (Supplementary Figure S2E). Therefore, Oct4 binds to LIN28B mainly through sequence specific binding of the POU_HD_ to the HD^–7^ site.

Reconstitution of ESRRB WT and mutant sequences resulted in an assembly doublet (Figure 1F, Supplementary Figure S1F), which may be explained by the co-existance of two predominant conformations. Assemblies were routinely heat-shifted following salt-gradient dialysis suggesting both populations are thermodynamically stable (see Materials and Methods). Equal nucleosome-histone stoichiometry was also checked on coomassie stained SDS-polyacrylamide gels and histones appeared in comparable proportions, suggesting the presence of conformational isoforms (Supplementary Figure S1E). Furthermore, adding increasing amounts of free H2A/H2B dimer to histone octamer during assembly had no effect on the presence of either band, suggesting neither band contains a hexasome, which could be formed by the eviction of one H2A/H2B dimer from the octamer in the nucleosome core (50). Footprinting demonstrated not only were assemblies comparable between sequences but also suggested the presence of a middle positioned nucleosome (Supplementary Figure S1G, H).

In contrast to LIN28B, ChIP-Seq data show ESRRB contains one clear Oct4 binding peak at a canonical POU_S_ half-site followed by a potential POU_HD_ binding site (Figure 1B, D). To confirm this binding site, we first mutated the POU_S_ site, S^+5.5^, and observed a complete loss of Oct4 binding to both the nucleosome and free DNA (Figure 1F, WT and 1mS^+5.5^). At high concentrations of Oct4, both free DNA and nucleosomes shifted to a smear rather than a defined band, which may be due to nonspecific interactions. When we integrated a homeodomain half-site directly 3′ to the mutated POU_S_ site, binding was not restored to either free DNA or the nucleosome (Figure 1F, 1mS^+5.5^+HD^+6^). This may be due to either the position of the POU_HD_ facing into the histone core or the POU_HD_’s inability to form a stable complex without the POU_S_ binding to its binding site. When we generated atomistic models of the ESRRB nucleosome we observed the POU_S_ half-site facing away from the histone core suggesting it would be accessible for Oct4 binding (Figure 1D). To check the potential binding of the POU_HD_ on an exposed position at SHL +5.5, we introduced a canonical homeodomain half-site in the POU_S_ half-site location (Figure 1B, F, +HD^+5.5^). This mutation abolished Oct4 binding to the nucleosome and allowed weak but distinct binding of Oct4 to free DNA, suggesting that both DNA sequence and structure play a role in nucleosome binding by the POU_HD_. Therefore, Oct4 binds to ESRRB at S^+5.5^ and the binding is driven by the POU_S_ domain.

### Oct4 uses nucleosome structural flexibility to bind

Recently, we reported differences in local nucleosome dynamics that extended beyond the linker DNA arms into the body of the nucleosome (27,36). Notably, dynamics were sequence dependent and greater in native nucleosomes, compared to the synthetic Widom 601 positioning sequence (34). We wanted to know how restricting these nucleosome dynamics would influence Oct4 binding.

**Figure 2.**
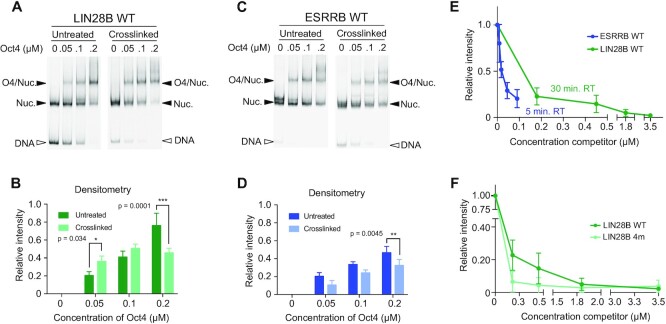
Nucleosome dynamics influence Oct4 binding. (A, B) LIN28B. (C, D) ESRRB. EMSAs of purified Oct4 with untreated (left) or crosslinked (right) nucleosomes (**A**, **C**). Filled arrowheads signal nucleosomes or nucleosome-protein complexes and empty arrowheads point to free DNA. The bar graphs display the mean and standard deviation of densitometry relative to starting nucleosome amounts (**B**, **D**). *n* = 3 per condition, * *P*= 0.0344,** *P*= 0.0045, *** *P*= 0.0001. (**E**) Densitometry of Oct4 competition EMSAs using LIN28B (green) or ESRRB (blue) WT nucleosomes (mean values with the standard deviations as error bars). For ESRRB *n* = 4, for LIN28B *n* = 5. (**F**) Same experiment as in panel E but comparing LIN28B WT and 4m nucleosomes (for 4m *n* = 6).

To test this, we crosslinked nucleosomes using formaldehyde (Supplementary Figure S3A) before performing binding assays. We performed densitometry on EMSA gels and show the ratio of Oct4-nucleosome complex relative to the amount of control nucleosome for each condition (Figure 2A–D). For LIN28B, crosslinking moderately increased binding at low Oct4 concentrations, but reduced binding at high concentrations (Figure 2A, B). Crosslinking ESRRB resulted in overall reduced Oct4 binding (Figure 2C, D). This suggests that the intrinsic dynamics of ESRRB facilitate Oct4 binding, while binding to LIN28B is more nuanced due to the presence of multiple Oct4 binding sites.

We then wanted to know how the different WT and mutated sequences affect the stability of the Oct4–nucleosome complexes. After protein–nucleosome complex formation, we added increasing amounts of unlabeled specific competitor and monitored Oct4’s dissociation from the nucleosome. Notably, Oct4 separation from the LIN28B nucleosome required 20–40 fold higher amounts of competitor and six times longer incubation than from the ESRRB nucleosome (Figure 2E, Supplementary Figure S3B). These results corresponded with the number of Oct4 binding sites on each sequence, the higher affinity of the POU_HD_ to free DNA comparing to POU_S_ (37), and the higher structural flexibility of the LIN28B nucleosome (36).

When we mutated all proposed Oct4 binding sites except the primary HD^-7^ site (‘4m’), the complex dissociated faster than the complex with the WT LIN28B nucleosome (Figure 2F, Supplementary Figure S3C). This shows that the mutated sites contribute to the affinity of Oct4 for LIN28B together with the primary HD^-7^ site. In summary, Oct4 has a higher affinity for the LIN28B nucleosome in part due to the multiple binding sites and the dynamics between the DNA and the histone core. Oct4’s affinity for the lone ESRRB nucleosome binding site is partially dependent on nucleosome dynamics and substantially lower than that for LIN28B (also seen from the apparent Kd values in Supplementary Figure 2E).

### Oct4 modulates nucleosome structural flexibility

**Figure 3.**
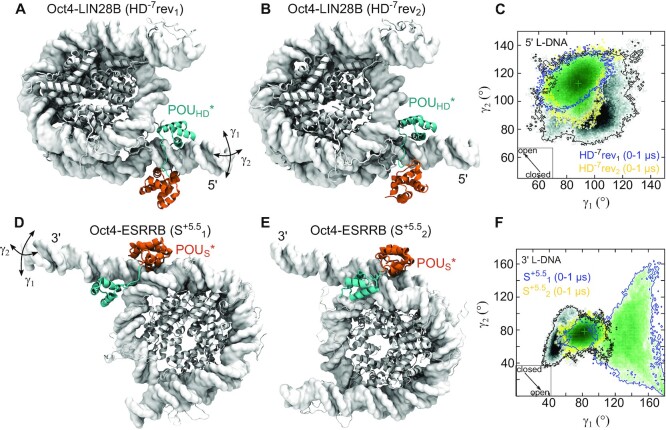
Oct4 binding modifies nucleosome breathing. (**A–C**) Oct4-LIN28B. (**D–F**) Oct4-ESRRB. Representative structures were selected from two independent simulations of each complex (A, B, D, E). In Oct4–LIN28B, Oct4 is bound to the HD^–7^ binding site, in reverse orientation (A, B). The configuration of Oct4 is either canonical (A) or generated from a simulation of apo Oct4 (B). In Oct4-ESRRB, Oct4 is bound to the S^+5.5^ site (D, E). Both starting configurations of Oct4 were taken from the simulation of apo Oct4. (see Materials and Methods). The structures were selected to correspond to the most sampled conformation in the following 2D histograms. The 2D histogram depict the γ_1_/γ_2_ conformational sampling in two simulations (2 μs aggregate time) of the free nucleosome (black) and two simulations (2 μs aggregate time) of Oct4-nucleosome complexes (green scatter plot with blue and yellow contours for the individual simulations) (C, F). The motions described by γ_1_ and γ_2_ are indicated with double headed arrows. The arrows in the square insets indicate the direction of the nucleosome opening. The ‘*’ labels the sequence specific bound subdomain (also in subsequent figures).

To characterize the structural basis for the Oct4-nucleosome recognition and to evaluate how Oct4 binding impacts nucleosome dynamics, we generated structural models of Oct4 bound to the different binding sites (Figure 3, Supplementary Figure S4), performed multiple MD simulations with these models (Table 1), and compared them with the published simulations of free nucleosomes (27,36). We quantified nucleosome dynamics by measuring overall nucleosome compactness using the radius of gyration (*R*_g_) and the breathing motions in two orthogonal planes, the plane of the core histones in a 2D top view of the nucleosome (angle γ_1_) and a plane perpendicular to it (angle γ_2_) (27,36,47) (see Materials and Methods).

First, we extended the simulations of Oct4 bound to the sites proposed by Soufi *et al.* (11) on LIN28B (27) to 1 μs. Oct4 binding remained stable to these sites on this time-scale (Table 1) and did not influence the nucleosome dynamics (Supplementary Figure S4A,B).

To model Oct4 bound to the HD^–7^ site without clashes with the inner DNA gyre, we had to use partially open conformations of LIN28B from the simulations of the free nucleosome. We modelled two binding orientations as for the HD^–4.5^ site (27). The ‘reverse’ orientation has the POU_HD_ bound to the typical homeodomain site TAAT(AC) on the 3’-5’ genomic DNA strand. The ‘forward’ orientation has the POU_HD_ bound to the GTAT(TA) motif on the genomic 5’-3’ DNA strand (Table 1, Supplementary Table S1). The globular region of the POU_HD_ recognizes specifically the central AT bases in both orientations. In contrast to the HD^-4.5^ site on which Oct4 binding was stable only on the forward orientation (27), on the HD^-7^ site, Oct4 binding was stable in both orientations (Videos S1, S2). Because the two orientations have a similar effect on the nucleosome structural flexibility, we only present the data for the reverse orientation. The data for the forward orientation is available in the Supplementary Materials (Supplementary Table S1, Supplementary Figures S4, S5).

The HD^-7^ site is located in the L-DNA but near the inner gyre (Figure 1C). This allows Oct4 binding in the canonical configuration (Table 1, Figure 3A). To sample alternative configurations with the POU_S_ interacting nonspecifically with the nearby DNA segments, we also built models of the complex with MD-generated Oct4 configurations (Figure 3B). The binding of Oct4 was stable on the 1μs timescale and maintained the LIN28B nucleosome in partly open conformations, rarely sampled in simulations of the free nucleosomes. Practically, the position of the sequence-specific bound POU_HD_ between the two DNA gyres blocked LIN28B closing (Figure 3C, Supplementary Figure S4).

In contrast, the binding of Oct4 in the canonical configuration was not possible on the ESRRB nucleosome because the binding site is located in the core nucleosomal DNA (Figure 1D). Instead, we modelled the Oct4-nucleosome interaction using alternative, MD-generated Oct4 configurations (Table 1, Figure 3D,E, see also Methods).The DNA readout of the POU_S_ domain was maintained in all simulations analysed, indicating stable binding.

In one simulation of the Oct4–ESRRB complex (S^+5.5^_1_) the nucleosome opened with an amplitude significantly larger than the breathing amplitude of free nucleosomes on the same time scale (Table 1, Figure 3F, Supplementary Figure S6A and Video S3). In the other simulations, the sampled nucleosome conformations were similar to those observed in the simulations of the free nucleosome (Supplementary Figure S6A and Video S4). The 2D γ1/γ2 histograms from all simulations of Oct4-ESRRB complex (Table 1, Supplementary Table S1) are shown in Supplementary Materials (Supplementary Figures S6, S7).

### Oct4 cooperates with histone tails to unravel genomic nucleosomes

Next, we wanted to know whether the differences between the simulations of the Oct4-ESRRB complex are due to the histone tails, which are known to regulate inter- and intranucleosome dynamics (36,51). For this, we characterized how Oct4 binding influences the interaction of histone tails with the DNA by calculating the number of stable tail–DNA contacts (defined as contacts present in at least 75% of the simulation). We focused on those tails in the proximity of the HD^–7^ and S^+5.5^ binding sites on LIN28B and ESRRB, respectively (one monomer of H3, H2AC, H4 and H2B), and compared the simulations with and without Oct4 bound.

**Figure 4.**
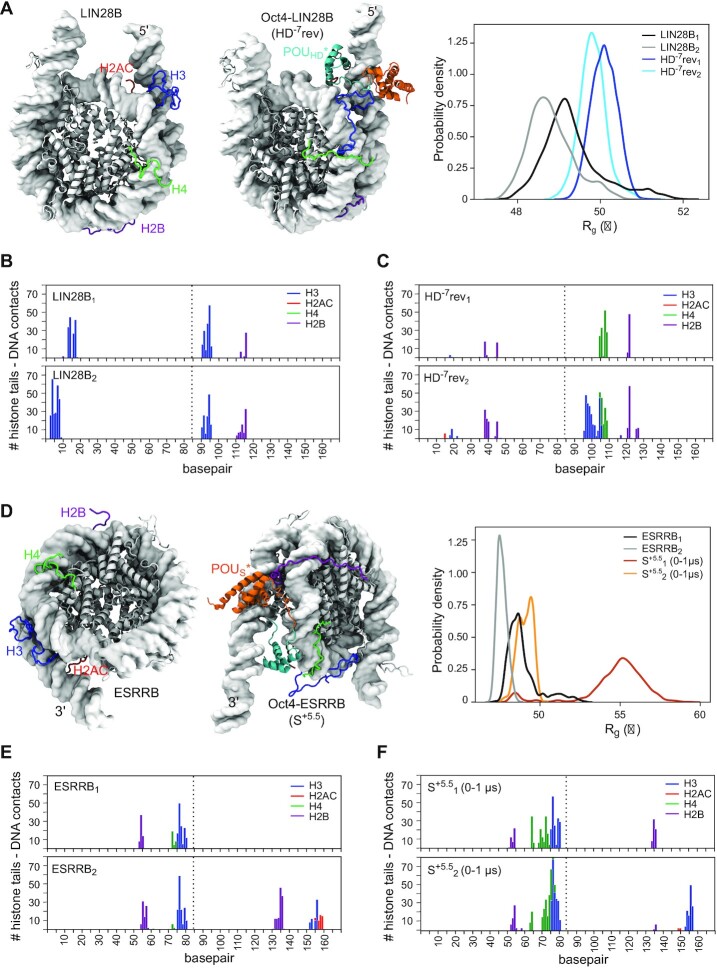
Oct4 modifies the histone tail-DNA interaction profiles.(**A–C**) LIN28B, free or with Oct4 bound to HD^–7^ in reverse orientation. (**D–F**) ESRRB, free or with Oct4 bound to S^+5.5^. Representative structures of the free nucleosome and Oct4-nucleosome complexes are shown together with the *R*_g_ histograms (A, D) to illustrate the nucleosome conformations sampled. The histone tail–DNA interaction profiles show the number of stable contacts between the tails in the proximity of the Oct4 binding site and the DNA in the simulations of free nucleosomes (B, E) and Oct4–nucleosome complexes (C, F). A contact was defined as stable if it was present in more than the 75% of the 1 μs simulation time. The dotted line marks the position of the dyad

We previously reported that free LIN28B nucleosome opening was facilitated when the H3 and H2AC tails established fewer interactions with the L-DNA and more interactions with the core DNA (36) (Figure 4A, B). When Oct4 was bound to the main HD^-7^ site (Figure 4A, C, Supplementary Figure S5A, B), the nucleosome mostly sampled partially open conformations (Figure 4A) with very few stable contacts between H3 and the 5’ L-DNA (Figure 4C, Supplementary Figure S5A, B). The position of the POU_HD_ bound at the edge between the L-DNA and the core nucleosomal DNA and the Oct4 linker in the canonical configuration (HD^-7^rev_1_ simulation), together blocked H3 tail binding to L-DNA and near the dyad region (base pairs 90–95) (Figure 4A, C). Moreover, the binding of Oct4 to the S^–1^ but not HD^–4.5^ site also resulted in fewer stable contacts between histone tails and the DNA (Supplementary Figure S5C, D).

Both in the ESRRB alone and the Oct4-ESRRB complex the nucleosome remained closed (Figure 4D) in all simulations in which stable interactions were formed between the H3 and H2AC tails and the DNA at the 3’ end of the nucleosome (Figure 4E,F, Supplementary Figure S8A).

Independent of the DNA sequence and the location of the binding site, the number of stable contacts between the H4 tail and the DNA increased upon Oct4 binding (Figure 4C, F, Supplementary Figures S5, S8). While this had no significant impact in nucleosome dynamics, it may affect the inter-nucleosome stacking in the presence of Oct4 since H4 tail is known to play a role in these interactions (52–54).

**Figure 5.**
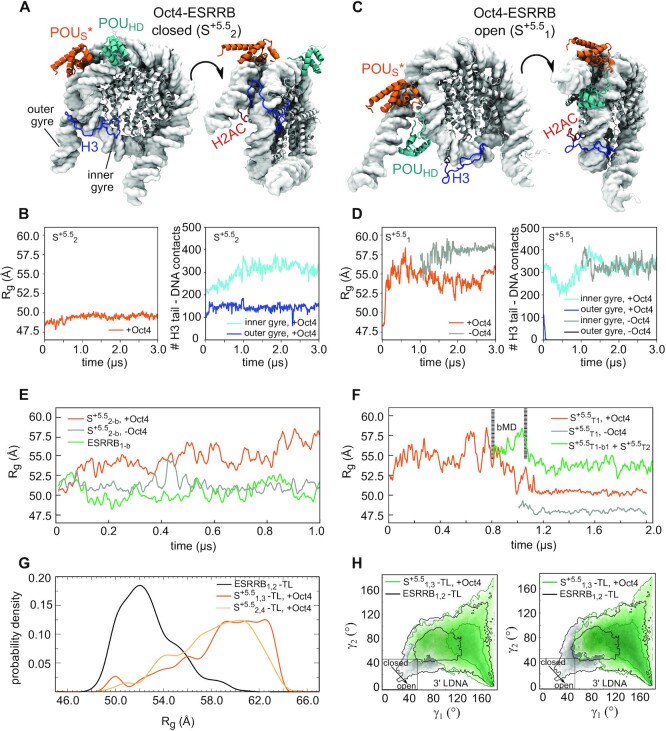
Oct4 stabilizes and enhances histone tail mediated nucleosome opening (A, B) Oct4–ESRRB complex with the nucleosome closed. (C, D) Oct4–ESRRB complex with the nucleosome open. Representative structures from the S^5.5^_2_ (**A**) and S^5.5^_1_ (**C**) simulations are shown together with the corresponding time series of *R*_g_ (orange) and the number of contacts between the H3 tail and the inner (cyan) and outer (blue) gyres of the DNA (**B**, **D**). In gray/black are data from a simulation started after 1 μs of S^5.5^_1_ after removing Oct4. (**E**) ESRRB nucleosome opening in simulations biased to prevent interactions between the H3 and H2AC tails near the Oct4 binding site and the outer DNA gyre. Time series of *R*_g_ are shown from simulations started after 1 μs of S^5.5^_2_ with (orange) or without Oct4 (grey) and after 1 μs of ESRRB_1_ (green) (see Methods). (**F**) ESRRB nucleosome opening in simulations started with the H3 and H2AC tails remodelled using a representative configuration selected from the open nucleosome conformation found in S^5.5^_1_. Time series of R_g_ from three simulations are shown: S^5.5^_T1_ (orange), (S^5.5^_T1_–Oct4) started after 1 μs of S^5.5^_T1_ after removing Oct4 (grey), and a 1 μs unbiased simulation (green) started after 800 ns of S^5.5^_T1_ and 250 ns in which a bias was added (S^5.5^_T1-b1_) to move the POU_HD_ in between the two DNA gyres (see Materials and Methods). (F, G) ESRRB nucleosome opening in simulations started after removing the histone tails (tail-less ESRRB-TL nucleosome). Histograms of *R*_g_ (**G**) and γ_1_/γ_2_ angles (H) were calculated from 4 μs ensembles (two independent simulations, each 2 μs long). The ensemble of the free ESRRB-TL (black) is compared with two ensembles of Oct4-ESRRB-TL complexes (dark and light orange in (G), green in (**H**)) which differ in the configuration Oct4 used to start the simulations (Table 1). Two contours are shown in the 2D histograms corresponding to 1 and 100 counts.

When ESRRB remained closed with Oct4 bound, the H3 and H2AC tails interacted with both the inner and outer gyres of the DNA (Figure 5A, B). When ESRRB opened, these interactions were absent and the open conformation was stable even after removing Oct4 (Figure 5C, D). Based on these findings, we hypothesized that the large amplitude opening occurred only in a single simulation of the Oct4–ESRRB complex because of the limited sampling of the mobility of histone tails on the 1μs timescale (55,56). In most simulations, the tails collapsed on the DNA and tail–DNA binding/unbinding events required for nucleosome opening were very rarely sampled. If our hypothesis was right, minimizing the interactions between H3 and H2AC tails and the outer gyre of DNA should facilitate nucleosome opening.

To test this, we took three different approaches. First, we performed MD simulations in which the H3 and H2AC tails were biased not to interact with the outer gyre of the DNA. For this, we added harmonic biases to the minimal tail-DNA interatomic distances and to the corresponding coordination numbers, which reflect the number of contacts (see Methods). We started simulations from two of the simulations in which the nucleosome remained closed (S^+5.5^_2_ and S^+5.5^_3_), with and without Oct4 present. We also started biased simulations from the classical simulations of the ESRRB alone (Table 1, Supplementary Table S1). Remarkably, ESRRB opened in all biased simulations and the amplitude of the opening was larger with Oct4 bound (Figure 5E, Supplementary Figures S6B–D, S8C, S9A, Supplementary Video S5). Moreover, the open conformations were stable in 1μs of unbiased simulations, started after the biased simulations (Supplementary Figure S9B, C). In all biased simulations, the harmonic wall biases were effectively active only at the beginning of the simulations. In most simulations of the Oct4–ESRRB complex, once ESRRB opened it did not close back, suggesting that the bias only enhanced a natural motion of the Oct4-bound nucleosome.

Second, we performed a classical simulation of the Oct4–ESRRB complex (S^+5.5^_T1_) starting with a partially closed nucleosome but with configurations of the H3 and H2AC tails selected from the open ESRRB conformation established in the simulation S^+5.5^_1_ (Table1). Again, the nucleosome opened (Figure 5F, Supplementary Figures S7A, S8D Video S6). However, after 1μs, the nucleosome closed into a compact conformation (Figure 5F, orange). This conformation was even more compact in a simulation started after 1μs of S^+5.5^_T1_ after removing Oct4 (Figure 5F, grey).

Third, we performed simulations with tail-less (‘-TL’) nucleosomes. As expected, as we’ve previously shown, the breathing motions of the free ESRRB-TL nucleosome had a larger amplitude than those of the complete nucleosome (36). However, in the presence of Oct4, the opening of the ESRRB-TL nucleosome was much larger (Figure 5G,H, Supplementary Figure S9D-F).

In summary, these data shows that Oct4 stabilizes and further enhances the opening of ESRRB. However, in native, complete nucleosomes, the impact of Oct4 on nucleosome dynamics depends on the mobility of the H3 and H2AC tails.

### Oct4’s subdomains have distinct roles in stabilizing open nucleosomes

Comparing two classical simulations of the Oct4-ESRRB complex in which ESRRB opened extensively (S^+5.5^_1_ and S^+5.5^_T1_), we observed in the first that the open conformation was not only stable for 3μs in the presence of Oct4 but it also remained stable when Oct4 was removed (Figure 5D, Supplementary Figure S6A), whereas in the second, the nucleosome closed after 1 μs (Figure 5F, Supplementary Figure S7A). In the first simulation, the open conformation was stabilized by nonspecific interactions between the POU_HD_ and both DNA gyres, which prevented the nucleosome from closing (Figure 5C). We confirmed this by performing two 250 ns biased MD simulations started after 800 ns of S^+5.5^_T1_ (at maximum opening). In these, we biased the POU_HD_ to move in between the gyres using harmonic steered MD biases while the nucleosome was maintained open with harmonic wall biases preventing interactions between the H3 and H2AC tails and the outer DNA gyre (see Methods). After 250 ns of each simulation, we switched off the bias for 1 μs of classical simulation (S^+5.5^_T2_, S^+5.5^_T3_) (Table 1). In both these simulations, the nucleosome remained open (Figure 5F, Supplementary Figures S7, S8D, S9G, Supplementary Video S7).

**Figure 6.**
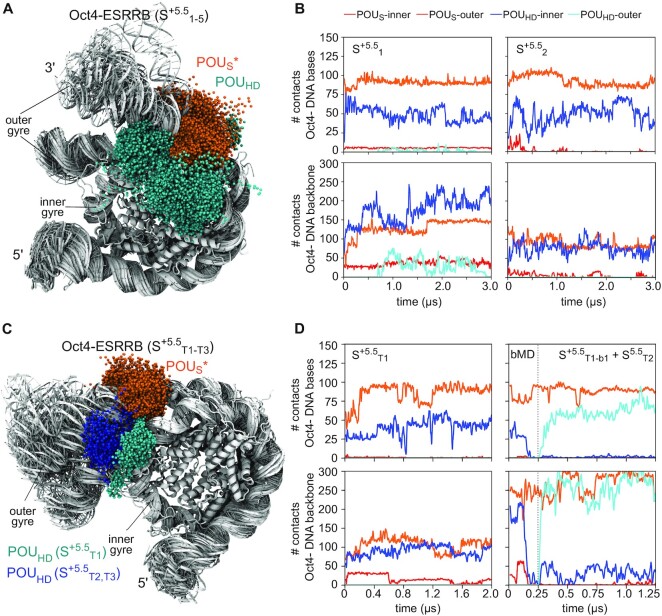
Oct4 modifies nucleosome dynamics by sequence specific DNA recognition with one subdomain and nonspecific DNA interactions with the other subdomain. (**A**) Sampling of the nucleosome surface by the Oct4 subdomains in 10 μs aggregate simulation time of the Oct4-ESRRB complex. (**B**) Number of contacts between the POU_S_ (red, orange) or the POU_HD_ (blue, cyan) with DNA bases (sequence specific, upper plots) or DNA backbone (nonspecific, lower plots) of the inner gyre (red, blue) or the outer gyre (orange, cyan) in two of the simulations shown in (A) (S^+5.5^_1_ and S^+5.5^_2_). (**C**) Same as (A) in 4 μs aggregate simulation time of the Oct4-ESRRB complex started with the H3 and H2AC tails remodelled using a configuration selected from the open nucleosome conformation found in S^+5.5^_1_. (**D**) Same data as in (B) but from the simulations S^+5.5^_T1,T2_.

Visualizing the position of the Oct4 subdomains during 10 μs aggregate simulation time of the Oct4-ESRRB complex (Figure 6A), we observed a narrow distribution for the position of the POU_S_, which forms sequence specific contacts to the outer gyre (Figure 6B). The position of the POU_HD_ showed a wider distribution with a large number of contacts both with DNA bases and the backbone of the inner gyre. When the ESRRB opened (simulation S^+5.5^_1_), its contacts with the inner gyre were maintained while new contacts were formed with the outer gyre (Figure 6A). Similar distributions were observed in the 4 μs aggregate simulation time of the Oct4-ESRRB complex started with H3 and H2AC configurations selected from an open nucleosome conformation (Figure 6C). In the first simulation (S^+5.5^_T1_) in which the nucleosome closed after 1μs, the POU_HD_ interacted only with the inner gyre, whereas in the other two, it formed nonspecific interactions with both gyres, preventing nucleosome closing (Figure 6D).

Moreover, we performed two additional 3 μs classical simulations, replicates of S^+5.5^_T1_ which provided further evidence for the POU_HD_-mediated stabilization of the open nucleosome conformation. In one (S^+5.5^_T4_ in Supplementary Table S1), ESRRB remained open with the POU_HD_ moving spontaneously between the two gyres, while in the other ESRRB closed like in S^+5.5^_T1_ (Supplementary Figures S7, S8D, S9H) because the POU_HD_ did not occupy the position between the gyres.

In contrast, the partially open conformation of the LIN28B nucleosome was stabilized by the sequence specific binding of the POU_HD_ to the outer gyre and by nonspecific interaction of the POU_S_ with the outer gyre (Supplementary Figure S10).

In summary, these data show that Oct4 uses both domains to bind to and stabilize open nucleosomes. With one of its subdomain it recognizes specific sequences and with the second it establishes barriers to nucleosome closing by nonspecific interactions with nearby DNA segments

## DISCUSSION

How pioneer factors (4,5) contribute to the transition of closed, silent chromatin to transcriptionally active DNA remains poorly understood. They bind to DNA wrapped in nucleosomes sometimes using only partial binding motifs and a range of translational and orientation binding preferences, suggesting a diverse range of potential pTF mechanisms (6). From the structural studies providing the first evidence of a direct TF-induced nucleosome opening, it has been proposed that for Oct4-Sox2 composite motifs, Sox2 binding to the minor groove deforms the DNA and mechanically loosens the grip of histones, freeing up buried binding sites and facilitating Oct4 binding (13,25). This mechanism involves Sox2-induced DNA opening and the use of only one of Oct4’s DNA binding subdomains, the POU_S_ domain. However, several questions remain unanswered: (i) Does the multi-domain pTF Oct4 alone have a similar impact on the nucleosome as Sox2?, (ii) How do pTFs bind native nucleosome sequences?, and (iii) How do histone tails modulate or adapt to the binding?

**Figure 7.**
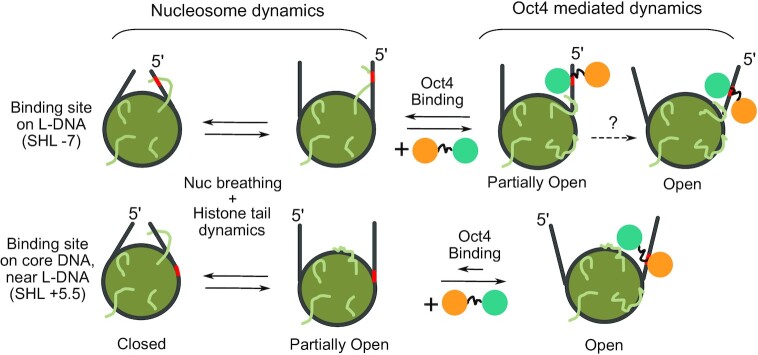
Summary of Oct4 mediated nucleosome dynamics The Oct4 POU_S_ and POU_HD_ subdomains are shown as orange and teal circles, respectively. Larger circles with straight lines represent nucleosomes and their linker DNA as viewed from the top in closed, partially open, or fully open configurations. The red points DNA indicate Oct4’s sequence specific binding half-sites. Coiled lines represent histone tails. Dotted arrows indicate hypothetical transitions.

Here we found that Oct4 can use either domain to recognize its binding sites on nucleosomes and we describe at atomistic resolution the interplay between intra-nucleosome dynamics and pTF binding (summarized in Figure 7).

Oct4 binds to one native nucleosome, LIN28B, primarily on a POU_HD_ binding site at the 5’ end of the nucleosomal DNA (the HD^-7^ site), but also to the POU_S_ and POU_HD_ sites located in the core nucleosomal DNA (11). From our experiments, the affinity of Oct4 for LIN28B appears lower than that measured by Soufi *et al.* (11). This is most likely due to differences in experimental setup (e.g. preparation of nucleosomes and Oct4 protein). Mutating only the HD^-7^ site reduced Oct4 binding substantially, whereas mutating the other binding sites had only a moderate effect, in agreement with other studies of Oct4 binding to the LIN28B nucleosome (11,12,14). Overall, these data point to Oct4’s adaptability as a pTF, as it is able to recognize multiple binding sites on the LIN28B nucleosome using either the POU_S_ or POU_HD_.

On another native nucleosome, ESRRB, Oct4 binds to the POU_S_ binding site positioned at SHL +5.5. The dissociation of Oct4 from this nucleosome was significantly faster than dissociation from LIN28B. This may be explained both by the difference in the number of binding sites between the two nucleosomes and by the higher affinity of the POU_HD_ domain to the DNA compared to the POU_S_ (9,37).

We turned to molecular modelling and simulations to reveal the structural basis of Oct4-nucleosome interaction at atomic resolution. These methods are reliable alternatives to experiments but depend on some approximations. For example, to build structural models of Oct4-nucleosome complexes we first had to build models of the free nucleosomes. For this, we positioned the dyad at the center of the reported micrococcal nuclease (MNase) peak and optimized the accessibility of the proposed Oct4 binding sites (10,27,36). This is an approximation as MNase-seq does not reveal the nucleosome position at base pair resolution. The cryo-EM structure of the free LIN28B, which was not available when we started our study, revealed that our approach provided reliable models. The structure differs from our models by just one base pair (26). This minimal difference can be attributed to the intrinsic flexibility of genomic nucleosomes (27,36). Moreover, the structure was resolved with 147 and not 168 base pairs of DNA, a difference that may also lead to minimal variations in positioning.

To validate our models of the Oct4-nucleosome complex, we performed short MD simulations after we fixed Oct4-DNA contacts in the equilibration phase. The contacts were selected from the crystal structure of Oct4 bound to free DNA (38). This assumed the same contacts are formed with the nucleosomes, which has been confirmed for the POU_S_ domain in the new structure of Oct4-Sox2-nucleosome complex (13). The question whether the homeodomain of Oct4 prefers a certain orientation to bind to nucleosome remains unanswered because for the HD^-7^ site we obtained stable Oct4 binding in both orientations.

We show that the intrinsic nucleosome flexibility is important for Oct4 binding. This may be particularly relevant for genomic nucleosomes as they are generally believed to be more flexible than those assembled on engineered sequences (57–59). Oct4 binding to crosslinked nucleosomes was reduced at high concentrations of Oct4. The moderate increase in the binding of Oct4 to crosslinked LIN28B at low Oct4 concentrations can be explained by the location of the predominant HD^-7^ on the linker DNA arm. We show that the binding of Oct4 to this higher affinity site requires more open conformations of LIN28B, which LIN28B often adopts (27,36). When crosslinked, LIN28B nucleosomes are fixed in a range of conformations. At lower concentrations, Oct4 could predominantly bind to more open conformations with higher affinity, which then become saturated at higher Oct4 concentrations. Without crosslinking, the more open conformations are short-lived and Oct4 binds predominantly to partially open conformations on the HD^-7^ site but also to closed nucleosomes on the other binding sites with lower affinity. Another possibility is an induced fit mechanism in which Oct4 facilitates the opening of closed LIN28B conformations upon initial nonspecific binding. The sites with lower Oct4 binding affinity located on the nucleosome core are only occupied at higher Oct4 concentrations.

Moreover, we show that Oct4 not only stabilizes open nucleosome conformations but also induces nucleosome opening. When bound to the HD^-7^ site on LIN28B, Oct4 stabilized a partially open conformations by the sequence specific binding of the POU_HD_ in the space between the core and linker DNA (Figure 7). The POU_S_ domain interacted nonspecifically with the LIN28B DNA, sampling a range of positions. This suggests that one mechanism by which Oct4 impacts chromatin dynamics is by restricting the breathing of the nucleosome towards partly open conformations. Although we did not observe an Oct4 induced opening of LIN28B, we can’t exclude this as a possibility due to the limited sampling achieved in our simulations. When bound to the binding sites located in the core nucleosomal DNA, Oct4 did not affect the dynamics of LIN28B. Given that LIN28B is bound by several pTFs, it is also possible that nucleosome opening requires the binding of multiple TFs if the binding sites are located in the nucleosome core, as was recently proposed by an allosteric mechanism in which the binding of Sox2 affects Oct4 binding (14). Taken together, the current data suggest that the impact of Oct4 binding on nucleosome dynamics depends on the position of the binding site.

Using multiple approaches, including classical and biased simulations with complete and tail-less nucleosomes we revealed an Oct4-mediated opening of the ESRRB nucleosome. The open conformation was stable after Oct4 was removed and was conditioned by the mobility of the histone H3 and H2AC tails. The resulting open conformation was stabilized both by the sequence specific binding of the POU_S_ and nonspecific interactions between the POU_HD_ and both gyres of DNA, which prevented the closing of the nucleosomes. Interestingly, the intrinsic flexibility of free ESRRB was also higher at the 3’ end where the Oct4 binding site is located (36), suggesting that Oct4 is capable of mediating a large opening of the nucleosome only in regions with increased intrinsic flexibility.

The position and conformation of the H3 and H2AC tails are critical for nucleosome breathing (36,60,61) and Oct4 mediated opening. In general, binding and unbinding of histone tails from the DNA modulate intra-nucleosome dynamics. However, simulating this motion of the tails is the most challenging part of MD simulations involving nucleosomes and appears to depend not only on the time scale sampled but also on other factors such as the water and ion models and the force field used in the simulations (55,56). We propose that substantial, short lived opening of free nucleosomes as well as longer-lived Oct4-mediated opening is only possible when these tails establish a minimum number of interactions with the linker DNAs. As long as the tails embraced the L-DNA arms and bridged them to the core nucleosomal DNA, the nucleosome remained closed. When Oct4 occupied the HD^-7^ site on LIN28B, both tails adopted configurations with fewer contacts with the L-DNA arms. On the free LIN28B, these tails occupy the region where Oct4 binds, suggesting a competition between Oct4 and histone tail binding. However, for LIN28B to open and allow Oct4 binding, the tails need to free that region and interact with other pieces of DNA around it. We propose that Oct4 binds to pre-established partly open conformations in which the tails do not occupy the Oct4 binding region. Once bound, Oct4 can stabilize the partly open conformations by preventing the tails to occupy that region and close the nucleosome. The large opening of ESRRB was also only possible in the absence of H3 and H2AC interactions with L-DNA. Interestingly, in all our simulations, the binding of Oct4 to the nucleosome was accompanied by a substantial increase of the number of stable contacts between H4 tail and the nucleosomal DNA. Because the H4 tail is essential to establish nucleosome-nucleosome interactions (52–54), the binding of Oct4 might also alter the higher order chromatin structures. The interplay between TF binding and histone tails was also observed in the Sox2 bound nucleosome structure in which the H4 tail was displaced compared to the free nucleosome (25). Therefore, the interplay between pTF and histone tail binding to nucleosomal DNA is essential for nucleating the opening of nucleosome and ultimately chromatin structure.

In this interplay, the DNA-binding subdomains of Oct4 have distinct roles. One is recognizing specific DNA sequences while the other interacts nonspecific with nearby DNA segments. For sequence specific binding, the accessibility of the binding site is essential. One side of the DNA is occluded by the histone octamer and cannot be accessed unless the nucleosome unwraps. However, even when located on the accessible face of the DNA, the binding sites may still have different accessibilities depending on the location on the nucleosome relative to the dyad. For example, on LIN28B, Oct4 binds stronger to the HD^-7^ on L-DNA than to HD_-4.5_ on the core nucleosomal DNA although the sequences are identical. Moreover, on ESRRB, when replacing the specific binding site of the POU_S_ with a consensus POU_HD_ site, Oct4 did not bind, suggesting that in addition to sequence composition and accessibility, the binding is also determined by the local structure of the free nucleosome. Both Oct4 domains bind in the major groove and do not substantially alter the mechanical properties of the DNA structure, as other minor groove binders, such as Sox2, do (25). Therefore, we propose that Oct4 does not mediate nucleosome opening by modifying the local DNA structure but rather by altering the optimal wrapping of the two gyres around each other and the histones.

The Oct4 mediated opening is further stabilized by non-specific DNA interactions of the second subdomain which prevent the re-wrapping of the outer gyre, possibly propagating local chromatin opening. How often this happens in the genomic context remains unclear. This suggests that pTFs with multiple DNA binding domains use their domains not only to recognize the specific DNA sequence of a binding site but also to establish barriers to nucleosome closing or inter-nucleosome stacking by interacting with sequentially distant pieces of DNA. The relative motion of the domains in one TF is restricted by the linker between them. This may further explain why a major effect on nucleosome dynamics was observed only for certain binding site positions. For example, on LIN28B, the binding sites located in the nucleosome core are too far from the linker DNA arm to allow bridging of the two gyres by the Oct4 domains.

Finally, our study is a demonstration of the strength of molecular simulations in revealing the structural features and the dynamic mechanisms involved in pTF-nucleosome binding. On one hand, MD simulations are powerful tools to validate or exclude TF binding modes to nucleosomes (27). On the other hand, supported by direct experimental validation of Oct4 binding to different locations on different nucleosomes, MD simulations enabled the discovery of the elegant mechanism we present in this article by which Oct4 engages with and unravels nucleosomes. Many details of this mechanism are not accesssible from experiments. Although atomistic MD simulations have been extensively used to study nucleosomes structure, dynamics, and interaction (62), they are limited by the amount of sampling achieved (63), and coarse grained representations are currently needed for larger scale systems (64). Despite reporting here extensive aggregate simulation times, we did not observe multiple opening events in a single long simulation. This would be essential for obtaining a quantitative description of nucleosome dyanmics. However, Armeev *et al.* showed that even 15 μs are still not enough for observing such transitions in complete nucleosomes (65). In addition, the accuracy of the force fields used for simulating the dynamics of unstructured regions such as the histone tails is still under scrutiny (66,67). These limitations need to be addressed in future studies.

## DATA AVAILABILITY

All MD simulations presented (Table 1 and Supplementary Table S1) are available for download at the following link: https://datashare.mpcdf.mpg.de/s/OeXa432AsqwbGMC. See the ‘README_Huertas_MacCarthy_et_al_2022’ file therein for details and instructions.

## Supplementary Material

gkac755_Supplemental_Files
