# Supplementary material for: OCT4 interprets and enhances nucleosome flexibility
Source: Nucleic Acids Res. 2022 Sep 22;50(18):10311–27. doi: 10.1093/nar/gkac755 (PMC9561370; doi:10.1093/nar/gkac755)
Supplement: gkac755_Supplemental_Files [file gkac755_supplemental_files.zip › MacCarthy_Huertas_et_al_Supplementary_Materials_R.pdf]

MacCarthy and Huertas et al.: Oct4 interprets and enhances nucleosome flexibility

Supplementary Methods

DNaseI and restriction enzyme nucleosome footprinting

DNaseI footprinting - Cy5 3' single end-labeled nucleosomal DNAs were generated by PCR using the validated constructs from the section "Nucleosome reconstitution" as templates and then purified from 10% native gels by electroelution. Assemblies were reconstituted as described in the main methods section. Approximately 600 fmol of assembly or free DNA were partially digested with DNaseI (Thermo Fischer Scientific, USA) at 25°C for 2 minutes in EMSA binding buffer (25 mM HEPES pH 7.6, 50 mM NaCl, 0.5 mM EDTA, 1 μg/μL BSA, 0.8 mM DTT, and 10% glycerol) supplemented with $MnCl_2$. Reactions were stopped with the addition of an equal volume of Stop Buffer (2x - 90% deionized formamide, 0.1% bromophenol blue, 20 mM EDTA) and then heated to 95°C for two minutes. The entire reaction was run on 15% denaturing gels (1x TBE and 8 M urea) at 50 mA for 4 hours. Gels were imaged using a Fujifilm FLA-9000 fluorescence scanner (GE Healthcare).

Restriction digest footprinting - ESRRB free DNA and assemblies were digested overnight with equal amounts of AluI, HhaI, HinfI, and NcoI restriction enzymes (NEB, Ipswich, MA, USA) at 37°C. Reactions were then heated at 95°C for 10 min. to inactivate restriction enzymes as well as denature and dissociate histone proteins. Reactions were run on 20% native PA gels at 10 mA/gel for 3.5 hours and then imaged on Fujifilm FLA-9000 (GE Healthcare). The gels were then stained with Gel Red (Sigma Aldrich) and imaged on a UV transilluminator (VWR, USA).

Estimation of apparent equilibrium Oct4-nucleosome dissociation rates

Free and bound DNA bands were quantified using ImageQuant™ 7.1 TL (Cytiva) using constant lane width and band sizes across lanes. The fraction of bound free DNA or Nucleosome-DNA (F_B) were calculated for each protein concentration [P] where in (F_B = DNAbound/(DNAbound + DNAunbound). The equilibrium dissociation constant (K_d) was determined by non-linear curve fitting in R (http:// www.r-project.org/) using the equation below [1, 2] where: F_B = Fraction of DNA bound, K_d = Dissociation constant, [P] = total protein concentration, [D] = total DNA or nucleosome concentration:

$$F_B = \frac{[P]+[D]+K_d-\sqrt{([P]+[D]+K_d)^2-4[D][P]}}{2[D]}$$

Building the structural model of the 168 bp Widom nucleosomes

First, we built a 145 bp Widom nucleosome with full-length Drosophila histones, including tails. For this, we used Modeller (https://salilab.org/modeller) with the PDB structures 2PYO (Drosophila histones and human α-satelite DNA), 3LZ0 (*Xenopus Laevis* histones and Widom DNA) and 1KX5 (*Xenopus Laevis* histones and human α-satelite DNA) as templates. The DNA was transferred as a rigid body from the 3LZ0 structure, the core histones were modelled based on the 2PYO structure and the histone tails by homology modelling based on the 1KX5 structure (the only structure in which the tails are resolved). 100 models were generated using a "slow" optimization protocol followed by a "slow" MD-based refinement protocol (see Modeller user guide - https://salilab.org/modeller/10.3/manual/ - and references therein for these definitions). The model with the lowest discrete optimized energy was extended to 195 base pairs of DNA with the original Widom 601 sequence). First, the 3 base pairs at each end of each strand in the 3LZ0 structure were removed because they differ from the original Widom sequence. Then, fragments of 28 base pairs B-DNA generated with Nucleic Acid Builder, from Ambertools 18 were added to each end based on a sequence alignment between the DNA sequence from the 3LZ0 structure and the original Widom sequence. To optimize the computer resources needed for

the simulations, we kept only 168 bp DNA in our final model (11 and 12 bp L-DNAs at the 5'and 3' ends, respectively. To obtain this final model, we removed 13 and 14 base pairs from the 5' and 3' respectively from our 195 bp model. We used this model with Drosophila histones as template to built the equivalent model of a Widom nucleosome with human histones by homology modeling. Again, we built 100 models and selected the one with the lowest normalized DOPE score as defined in Modeller. These two models were then used as scaffolds to build the ESRRB and LIN28B nucleosomes as described in the main text.

Building structural models of Oct4-nucleosome complexes

For the models of the Oct4-LIN28B and Oct4-ESRRB complexes, we used the nucleosome with Drosophila and human histone, respectively. Our choice for the origin of the histones has a simple reason related to the chronology in which the experiments and the simulations were performed. First, we modelled and simulated the Oct4-LIN28B complex with Drosophila histones to match our initial experiments. Then, we modelled the Oct4-ESRRB complex with human histones because Soufi et al *(10,11)* performed their studies in human cells. While we performed experiments in parallel with both human and Drosophila histones, we found that the nucleosome assembly was more successful with the Drosophila histones. However, at this point the main set of the simulations of the Oct4-ESRRB complex with human histones were already in advanced stages. Therefore we continued the experiments with Drosophila histones and the simulations with the human histones. We expect the binding modes of Oct4 to nucleosomes to be independent on the histone sequences.
The sequences of the LIN28B and ESRRB used for the nucleosome models with the main binding sites (HD^{-7}, $HD^{-4.5}$, $S^{-1.5}$ in LIN28B and $S^{+5.5}$ in ESRRB) highlighted as in Figure 1 and the dyad in red are:

LIN28B (human genome assembly NCBI36 - hg18):
chromosome 6: 105,637,999-105,638,166

```
  1 AGTTAAGT GGTATTAA CATATCCT CAGTGGTG AGTATTAA CATGGAAC TTACTCCA
 57 ACAATACA GATGCTGA ATAAATGT AGTCTAAG TGAAGGAA GAAGGAAA GGTGGGAG
113 CTGCCATC ACTCAGAA TTGTCCAG CAGGGATT GTGCAAGC TTGTGAAT AAAGACAC
```

ESRRB (human genome assembly NCBI36 - hg18):
chromosome 14: 75,995,470-75,995,637

```
  1 ATCAGCAG GGAGAAGG AGCGCCTC CCCATGTG GGACCTGG AGAAACAG AGGGTGGA
 57 GGGAGCAT AGAGAGTC TGTTCTAA GCTGCAAA GCAAAGGC CTGGCGAC CTAGGAGA
113 CCATGGAG TTCCAGAA AGTGATAG TTATGCAG AGCGAATG GAGGGAAT CAGCACGC
```

The experimental data from where these were selected is available at GEO, with the accession codes GSE36570 (ChIP-Seq data) (*11*) and GSM543311 (MNase-Seq data) (*44*).

All models of the Oct4-nucleosome complexes presented in this manuscript were validated by monitoring the sequence specific and nonspecific interactions with of the subdomain of Oct4 that is bound specifically to each analyzed binding site. All the results we present here are from simulations in which the sequence specific contacts Oct4 with the nucleosomal DNA were preserved (see also (*19*)).

Equilibration protocol

The equilibration protocol was adapted from our previous simulations of Oct4 bound to the free DNA and is shown in the table below (see (*29*)).

Step	Time	Restraint type	Force constant (kcal/mol·A^2)	Restrained Atoms/Bonds*
1$^+$	150 ps	Positional	25	Protein and DNA heavy atoms
2	150 ps	Positional	10	Protein and DNA heavy atoms
3	250 ps	Positional	5	Protein and DNA heavy atoms
4	250 ps	Positional	1	Protein and DNA heavy atoms
5	250 ps	Positional<br>Distance	1<br>25	Protein and DNA backbone<br>DNA basepairs<br>OCT4-DNA base interactions
6	250 ps	Positional<br>Distance	1<br>10	Protein and DNA backbone<br>DNA basepairs<br>OCT4-DNA base interactions
7	250 ps	Positional<br>Distance	1<br>5	Protein and DNA backbone<br>DNA basepairs<br>OCT4-DNA base interactions
8	250 ps	Distance	1	DNA basepairs<br>OCT4-DNA base interactions
9	250 ps	Distance	0.5	DNA basepairs<br>OCT4-DNA base interactions
10	250 ps	Distance	0.1	DNA basepairs<br>OCT4-DNA base interactions
11	250 ps	Distance	0.05	DNA basepairs<br>OCT4-DNA base interactions
12	250 ps	Distance	0.01	DNA basepairs<br>OCT4-DNA base interactions
13	1.5 ns	-	-	
14	2.25 ns<br>(1.5 fs)$^\#$	-	-	
15	7 ns<br>(2 fs)$^\#$	-	-	

*Atoms for positional restraints, bonds for distance restraints.
$^+$ The first step includes the heating from 20 to 300K, and it's done in the NVT ensemble. All the other steps are done in the NPT ensemble at 300K.
$^\#$ The number in brackets indicates the timestep used in that step. For steps 1-13, a timestep of 1 fs is used. For the nucleosome alone systems, the protocol is identical, omitting the OCT4-DNA restraints.

Supplementary Data

- **Supplementary Table S1**: Overview of the simulations not included in the main manuscript.
- **Figure S1**: Characterization of *in vitro* reconstituted native nucleosomes (supplementary to data in Figures 1,2).
- **Figure S2**: Further experimental validation and characterization of the Oct4-nucleosome interaction (supplementary to data in Figure 1).
- **Figure S3**: Crosslinking and competition experiments (supplementary to data in Figures 2).
- **Figure S4**: Breathing motions of LIN28B in simulations of the Oct4-LIN28B complex with Oct4 bound to different sites (supplementary to data in Figure 3).
- **Figure S5**: Histone tail - DNA interaction profiles in the Oct4-LIN28B complex (supplementary to data in Figure 4).
- **Figure S6**: Breathing motions of ESRRB in classical and biased simulations of the ESRRB alone and the Oct4-ESRRB complex (supplementary to data in Figures 3, 5).
- **Figure S7**: Breathing motions of ESRRB in classical and biased simulations of the Oct4-ESRRB complex started with H3 and H2AC tail configurations selected from an open nucleosome conformation (supplementary to data in Figures 3, 5).
- **Figure S8**: Histone tail - DNA interaction profiles in the Oct4-ESRRB complex (supplementary to data in Figures 4, 5).
- **Figure S9**: ESRRB nucleosome opening in different simulations (supplementary to data in Figure 5).
- **Figure S10**: Sequence specific binding of the POU_{HD} and nonspecific interactions between the POU_{S} and the DNA on LIN28B (supplementary to data in Figure 6).
- **Video S1**: 1 μs simulation of Oct4 bound to LIN28B, in the "reverse" configuration ($HD^{-7}rev_1$). The starting Oct4 configuration was selected from a simulation of apo Oct4. Oct4 stabilizes a partially open nucleosome conformation.
- **Video S2**: 1 μs simulation of Oct4 bound to LIN28B in the "forward" configuration ($HD^{-7}for_1$). The starting Oct4 configuration was selected from a simulation of apo Oct4. Oct4 stabilizes a partially open nucleosome conformation
- **Video S3**: 2 μs simulation of Oct4 bound to the ESRRB nucleosome ($S^{+5.5}{}_1$). The starting Oct4 configuration was selected from a simulation of apo Oct4. Oct4 stabilizes an extensively open nucleosome conformation by sequence specific interactions between the POU_S and the DNA and by nonspecific interactions of the POU_{HD} with the two DNA gyres.
- **Video S4**: 1 μs simulation of Oct4 bound to ESRRB ($S^{+5.5}{}_2$). The starting Oct4 configuration was selected from a simulation of apo Oct4 (different than the one used in Video S3). The nucleosome remains closed due to the position of the unstructured tails of histones H3 and H2AC
- **Video S5**: 1 μs simulation Oct4-ESRRB complex in which a bias was applied to prevent interactions between the histone H3 and H2AC tails and the outer DNA gyre($S^{+5.5}{}_{2\text{-b}}$), followed by 1μs of unbiased simulation ($S^{+5.5}{}_{2'}$). The starting configuration was taken after 1μs of the $S^{+5.5}{}_2$ simulation. The nucleosome opens and then remains open.

- **Video S6**: 2 μs simulation of Oct4 bound to the ESRRB nucleosome ($S^{+5.5}{}_{T1}$). The starting Oct4 configuration was selected from a simulation of apo Oct4. The starting configurations of the H3 and H2AC tails were taken from the open conformation of the nucleosome found in the $S^{+5.5}{}_{1}$simulation. The nucleosome opens and Oct4 stabilizes this open nucleosome conformation. However, after 1 μs, the nucleosome closes because the POU_{HD} does not move into a position to interact with both DNA gyres, allowing the reformation of the interactions between the histone tails and the outer DNA gyre.

- **Video S7**: Biased simulations of Oct4 bound to the ESRRB nucleosome. 250 ns of a simulation where Oct4's POU_{HD} is biased to move between the two DNA gyres ($S^{+5.5}{}_{T1\text{-}b1}$), followed by 1$\mu$s of unbiased simulation ($S^{+5.5}{}_{T2}$). The starting configuration was the snapshot after 800ns of the $S^{+5.5}{}_{T1}$ simulation. The nucleosome remains open because POU_{HD} forms stable interactions with both DNA gyres as in the $S^{+5.5}{}_{1}$ simulation.

Bibliography

[1] Nithya BabuRajendran, Paaventhan Palasingam, Kamesh Narasimhan, Wenjie Sun, Shyam Prabhakar, Ralf Jauch, and Prasanna R. Kolatkar. Structure of Smad1 MH1/DNA complex reveals distinctive rearrangements of BMP and TGF-β effectors. *Nucleic Acids Res.*, 38(10):3477–3488, 2010.

[2] Daisylyn Senna Tan, Yanpu Chen, Ya Gao, Anastasia Bednarz, Yuanjie Wei, Vikas Malik, Derek Hoi Hang Ho, Mingxi Weng, Sik Yin Ho, Yogesh Srivastava, Sergiy Velychko, Xiaoxiao Yang, Ligang Fan, Johnny Kim, Johannes Graumann, Gary D. Stormo, Thomas Braun, Jian Yan, Hans R. Schöler, and Ralf Jauch. Directed Evolution of an Enhanced POU Reprogramming Factor for Cell Fate Engineering. *Mol. Biol. Evol.*, 38(7):2854–2868, 2021.

Table S1: Overview of the simulations presented in the Supplementary Materials

Simulation name[1]	DNA	Binding site	Starting structure[2]	Time	R_g[3]	Number of Oct4 - DNA contacts[4]			
						POU_S		POU_{HD}	
						Bases	Backbone	Bases	Backbone
$ESRRB_{2\text{-}b}$	ESRRB	-	-	1 μs	51.2 (49.5-52.7)	-	-	-	-
$HD^{-7}for_1$	LIN28B	HD^{-7}	Cano Oct4	1 μs	49.4 (49.0-49.9)	41 (23-54)	86 (63-109)	110 (93-132)	219 (179-260)
$HD^{-7}for_2$	LIN28B	HD^{-7}	MD Oct4	1 μs	49.6 (49.1-50.1)	3 (0-25)	27 (0-63)	88 (60-118)	157 (118-197)
$HD^{-7}for_3$	LIN28B	HD^{-7}	Cano Oct4	1 μs	50.0 (49.2-51.9)	46 (33-59)	110 (74-143)	88 (63-121)	184 (150-219)
$HD^{-7}for_4$	LIN28B	HD^{-7}	MD Oct4	1 μs	50.3 (49.7-50.9)	34 (1-55)	81 (18-111)	62 (43-86)	97 (64-157)
${S^{-1.5}}_1$	LIN28B	$S^{-1.5}$	MORE Oct4	1 μs	49.0 (48.4-49.5)	86 (69-102)	78 (63-93)	14 (6-39)	91 (51-129)
${S^{-1.5}}_2$	LIN28B	$S^{-1.5}$	MD Oct4	1 μs	48.8 (47.8-49.7)	82 (52-99)	81 (64-101)	2 (0-5)	29 (0-55)
$HD^{-4.5}for_1$	LIN28B	$HD^{-4.5}$	MORE Oct4	1 μs	49.7 (49.0-50.8)	31 (12-72)	92 (65-121)	51 (37-61)	92 (71-116)
$HD^{-4.5}for_2$	LIN28B	$HD^{-4.5}$	MD Oct4	1 μs	48.5 (47.9-49.3)	2 (0-8)	22 (3-48)	87 (71-104)	154 (116-193)
${S^{+5.5}}_{3\text{-}b}$	ESRRB	$S^{+5.5}$	1 μs of ${S^{+5.5}}_3$	1 μs	54.9 (50.0-56.5)	124 (103-150)	169 (129-235)	28 (14-41)	111 (77-151)
${S^{+5.5}}_{3\text{-}b}$ -Oct4	ESRRB	$S^{+5.5}$	1 μs of ${S^{+5.5}}_3$	1 μs	54.1 (50.8-55.6)	-	-	-	-
${S^{+5.5}}_{2'}$	ESRRB	$S^{+5.5}$	1 μs of ${S^{+5.5}}_{2\text{-}b}$	1 μs	55.8 (53.3-58.0)	94 (80-114)	172 (144-204)	79 (57-105)	63 (40-86)
${S^{+5.5}}_{2'}$ -Oct4	ESRRB	$S^{+5.5}$	1 μs of ${S^{+5.5}}_{2\text{-}b}$	1 μs	57.3 (55.1-59.6)	-	-	-	-
${S^{+5.5}}_{3'}$	ESRRB	$S^{+5.5}$	1 μs of ${S^{+5.5}}_{3\text{-}b}$	1 μs	55.5 (54.5-56.6)	137 (119-155)	221 (195-246)	23 (15-32)	108 (81-137)
${S^{+5.5}}_{3'}$ -Oct4	ESRRB	$S^{+5.5}$	1 μs of ${S^{+5.5}}_{3\text{-}b}$	1 μs	55.9 (54.8-57.8)	-	-	-	-
${S^{+5.5}}_{T4}$	ESRRB	$S^{+5.5}$	MD1 Oct4	3 μs	54.2 (52.9-56.1)	93 (78-120)	150 (116-203)	47 (7-82)	129 (70-193)
${S^{+5.5}}_{T5}$	ESRRB	$S^{+5.5}$	MD1 Oct4	3 μs	53.0 (50.3-54.8)	93 (78-107)	126 (88-171)	53 (34-78)	145 (88-181)

[1] ”T” = simulations started with H3 and H2AC tail configurations selected from open nucleosome conformations; ”b” = biased simulations.

[2] ”Cano”, ”MD” = Canonical and molecular dynamics based Oct4 configurations; ”nuc’ = nucleosome; ”eq” = equilibration.

[3] R_g and the number of contacts are shown as the median with the percentiles 5 to 95 in brackets.

[4] A contact was defined as a non-hydrogen atom closer than 4.5 Å to another non-hydrogen atom.

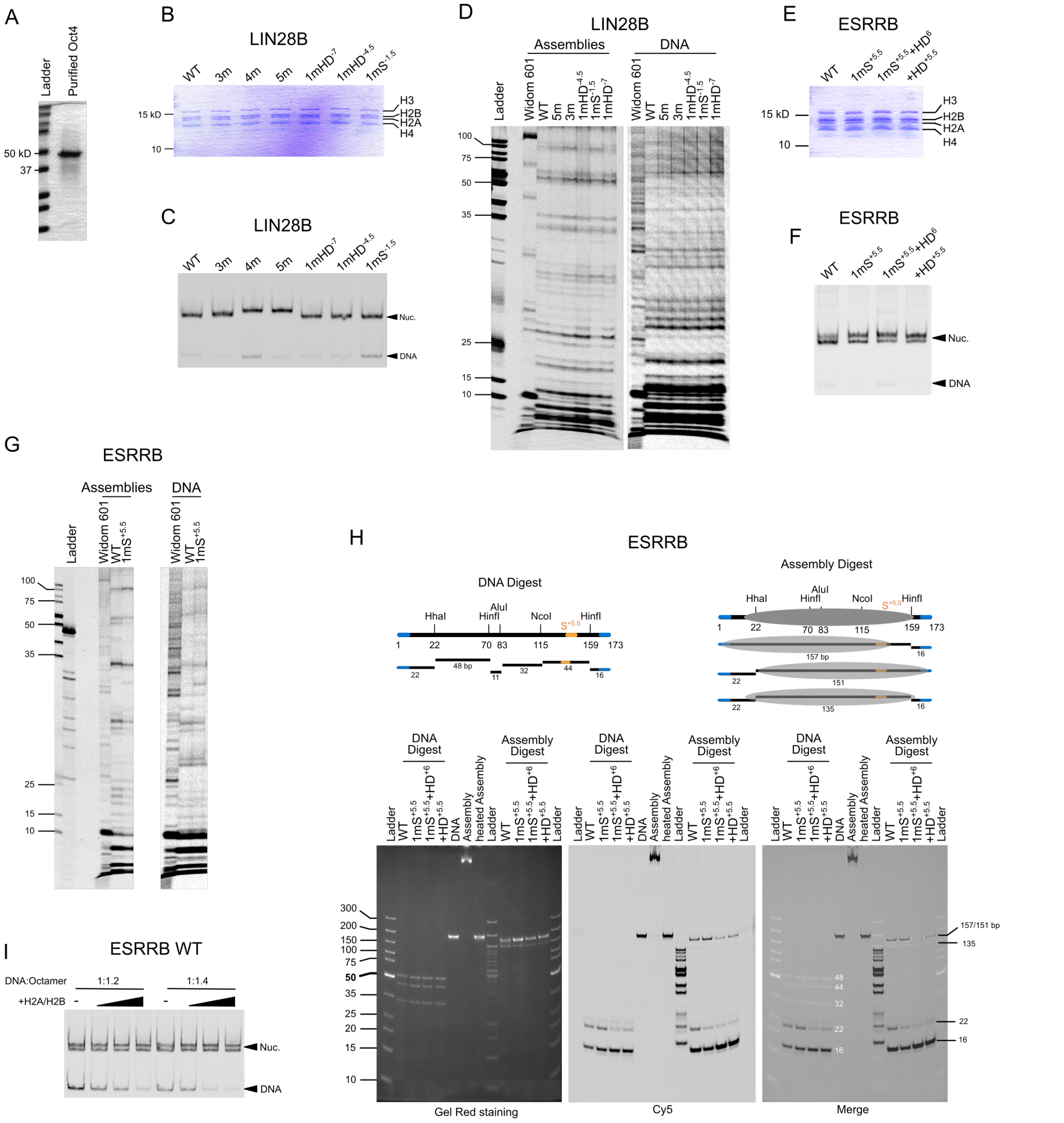

Figure S1: Characterization of *in vitro* reconstituted native nucleosomes. (**A, B, E**) Coomassie stained SDS-PA gels of (A) purified Oct4 protein (see main methods), (B) LIN28B nucleosomes, or (E) ESRRB nucleosomes. (**C, F**) Cy5 signal from native gels loaded with (C) LIN28B or (F) ESRRB nucleosomes. Arrowheads indicate assembled nucleosomes and free DNA. (**D, G**) DNase I footprints of (D) LIN28B or (G) ESRRB assemblies. (**H**) Gel red and Cy5 signals from ESRRB assembly restriction enzyme footprinting run on native gels. Top panels are schematic representations of free DNA or assembly digests. Blue and orange colors indicate Cy5 labeled ends and the POU_S half binding site ($S^{+5.5}$), respectively. Gray ovals represent the histone core position. Fragment length is indicated below fragments and on the merged gel. **I)** Cy5 signal from native gel loaded with ESRRB WT assemblies at two DNA:Octamer ratios supplemented with increasing amounts of purified H2A/H2B dimer. Arrowheads indicate assembled nucleosomes and free DNA.

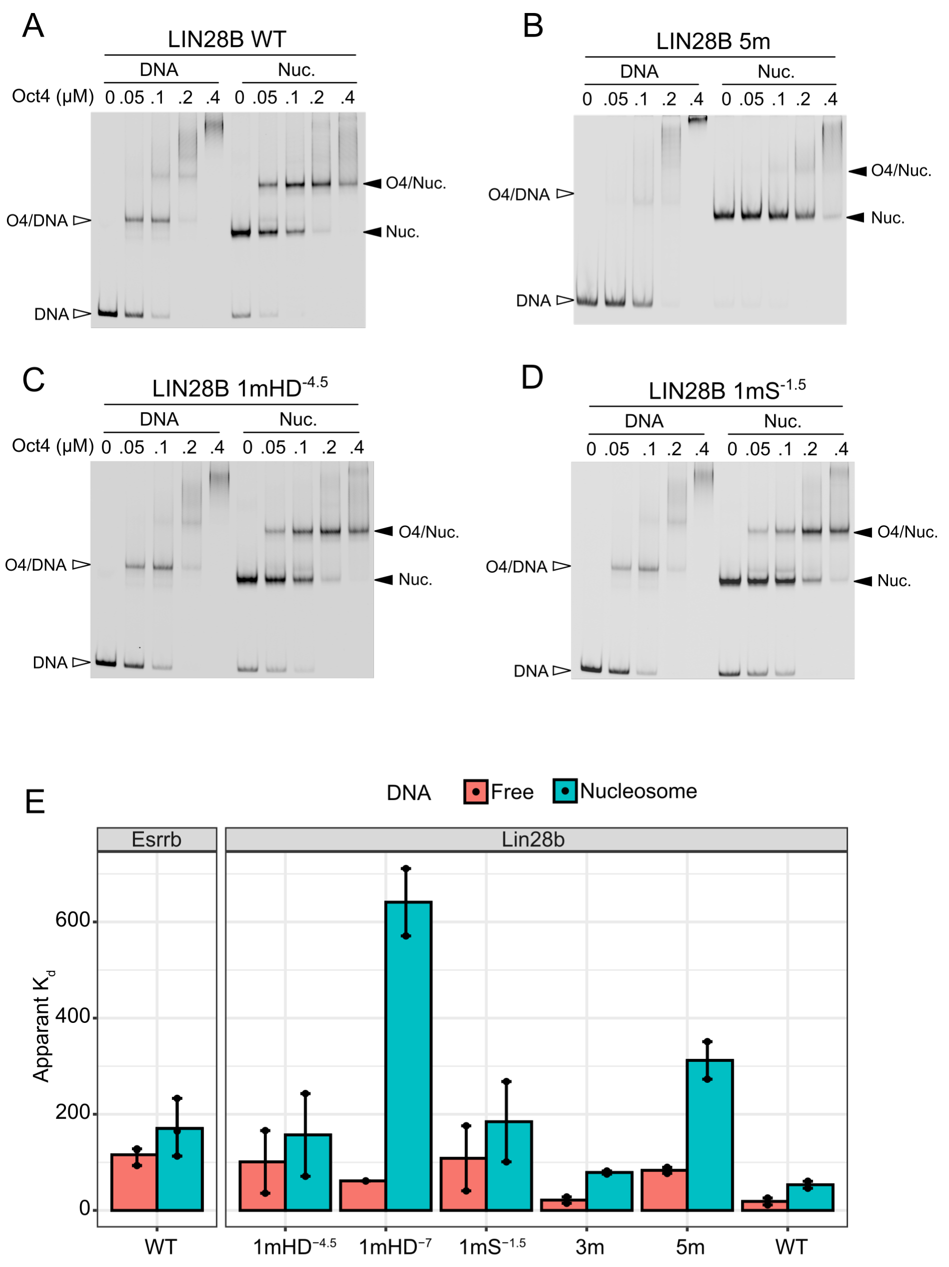

Sequence	Free DNA (nM)	Nucleosome (nM)
ESRRB	115.8	170.3
$1mS^{+5.5}$	nd	nd
$1mS^{+5.5}+HD^{+6}$	nd	nd
$+HD^{+6}$	nd	nd

Sequence	Free DNA (nM)	Nucleosome (nM)
LIN28B WT	18.6	53.4
3m	21.4	78.8
$1mHD^{-7}$	61.3	641
5m	83.3	312
$1mHD^{-4.5}$	100.9	157
$1mS^{-1.5}$	108.3	184.5

Figure S2: Further experimental validation and characterization of the Oct4-nucleosome interaction. (**A-D**) EMSAs of purified Oct4 with free DNA (left) and reconstituted nucleosomes (right). Filled horizontal arrowheads indicate nucleosomes or nucleosome-protein complexes and unfilled ones, free DNA or DNA-protein complexes. Wild-type and mutant sequences can be found in main Figure 1. (**E**) Estimation of apparent Oct4-nucleosome binding affinities. Apparent dissociation constant (K_d) values were calculated for total substrate bound as described in the supplementary methods section. Bar graphs represent the mean K_d values in nM for Oct4 binding to free DNA (red) or DNA incorporated into nucleosomes (teal) of two independent experiments for LIN28B sequences and three experiments for ESRRB. Whiskers indicate the data range. The data is also summarized in the tables underneath the bar plot. ESRRB mutant sequences did not furnish any discrete bands in EMSAs, thus K_d values for those could not be determined (nd).

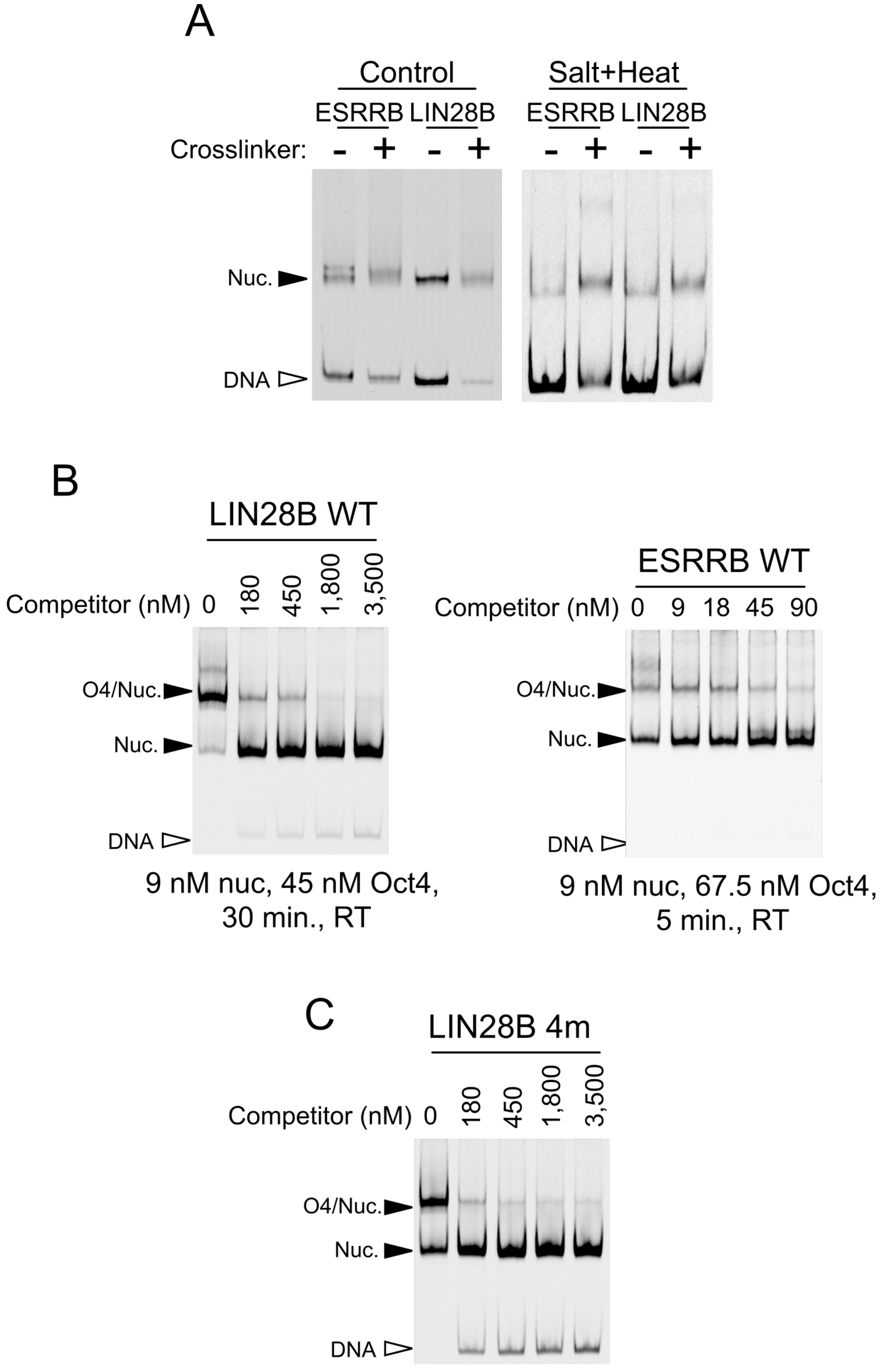

Figure S3: Crosslinking and competition experiments. (**A**)Native gels of LIN28B and ESRRB untreated and crosslinked nucleosomes. The right panel shows a representative native gel of a disassembly assay as a control for successful crosslinking. (**B,C**) Representative EMSAs of Oct4 competition experiments from Figure 2 panels E and F, respectively. Filled horizontal arrowheads indicate nucleosomes or nucleosome-protein complexes and unfilled ones, free DNA. See also Figure 2.

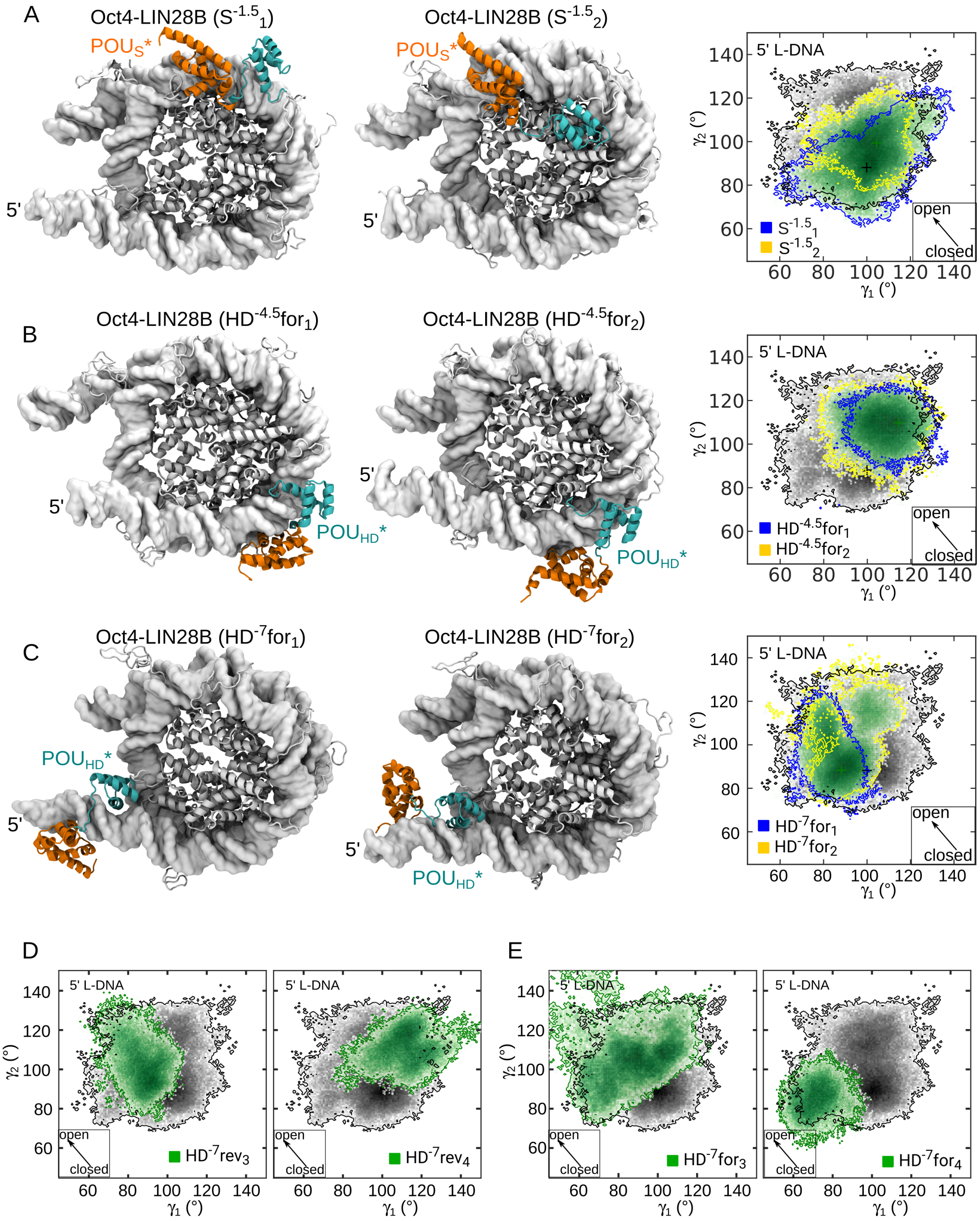

Figure S4: Breathing motions of LIN28B in simulations of the Oct4-LIN28B complex with Oct4 bound to different sites. (**A-C**) Representative structures and 2D histograms depicting the sampling of the γ_1/γ_2 conformational space from the simulations of Oct4 bound to LIN28B on the $S^{-1.5}$ site (A), $HD^{-4.5}$ site in forward orientation (B), and HD^{-7} site in forward orientation (C). (**D-E**) 2D γ_1/γ_2 histograms from additional simulations of Oct4 bound to LIN28B on the HD^{-7} site in reversed (D) and forward orientation (E). In the histograms, the sampling from the simulations of the free nucleosome is in black, whereas the sampling from the simulations of Oct4-LIN28B complex is in green. The yellow and blue contours indicate the sampling from the individual 1 μs simulations. The arrows in the square inserts indicate the direction of the nucleosome opening. The "*" labels the sequence specific bound subdomain. See also Figure 3.

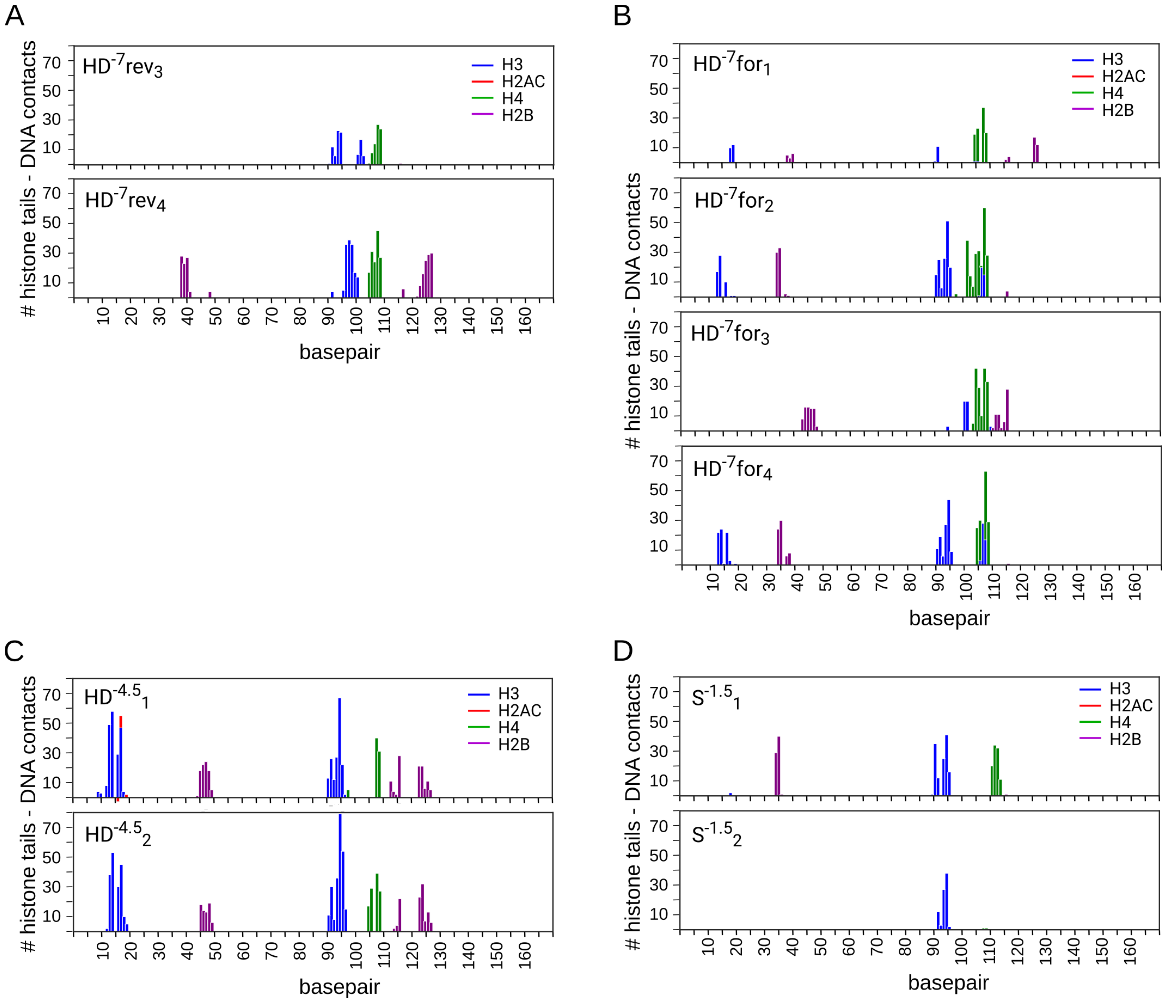

Figure S5: Histone tail - DNA interaction profiles in LIN28B and the Oct4-LIN28B complex. The number of stable contacts between the histone tails and the DNA is shown, with contacts defined as stable if they are present in more than the 75% of the simulation (as in Figure 4). (**A-B**) Additional simulations with Oct4 bound on the HD^{-7} site, in the reverse (A) and forward (B) orientations. (**C-D**) Simulations with Oct4 bound on the HD$^{-4.5}$ (C) and HD$^{-1.5}$ (D) sites. The data for H3, H2AC, H2B, and H4 tails are in blue, red, purple, and green, respectively. See also Tables 1, S1 and Figure 4.

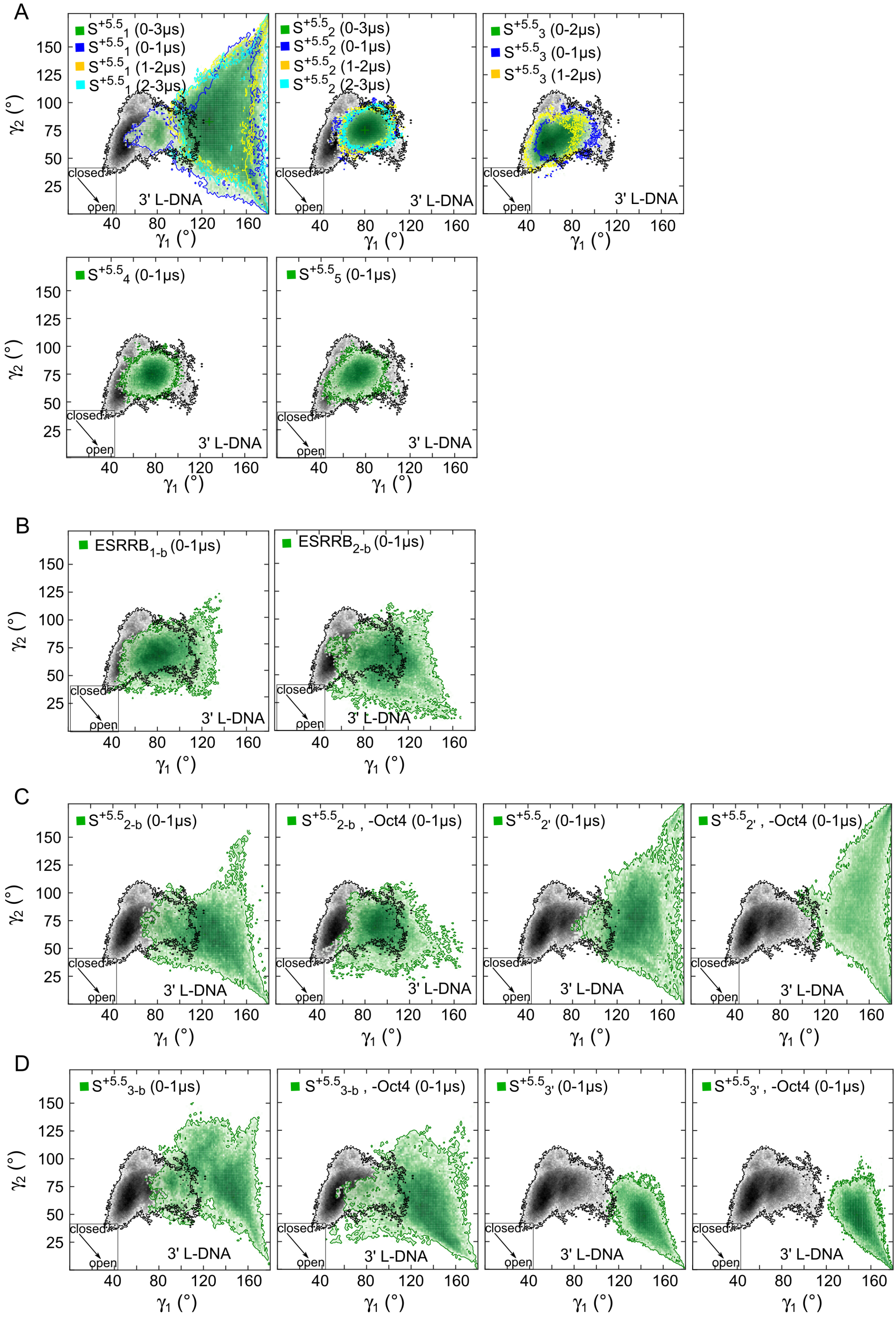

Figure S6: Breathing motions of ESRRB in classical and biased simulations of the ESRRB alone and the Oct4-ESRRB complex. (**A-D**) 2D histograms as in Figures 3 and S4 depicting the sampling of the γ_1/γ_2 conformational space from: the 5 classical simulations of the Oct4-ESRRB complex (A), the biased simulations of the ESRRB alone (B), the biased simulation ($S^{5.5}{}_{2\text{-b}}$) and the follow-up classical simulation ($S^{5.5}{}_{2'}$) of the Oct4-ESRRB complex started from the $S^{5.5}{}_{2}$ simulation (C), the biased simulation ($S^{5.5}{}_{3\text{-b}}$) and the follow-up classical simulation ($S^{5.5}{}_{3'}$) of the Oct4-ESRRB complex started from the $S^{5.5}{}_{3}$ simulation (D). See also Table 1 and Figure 3.

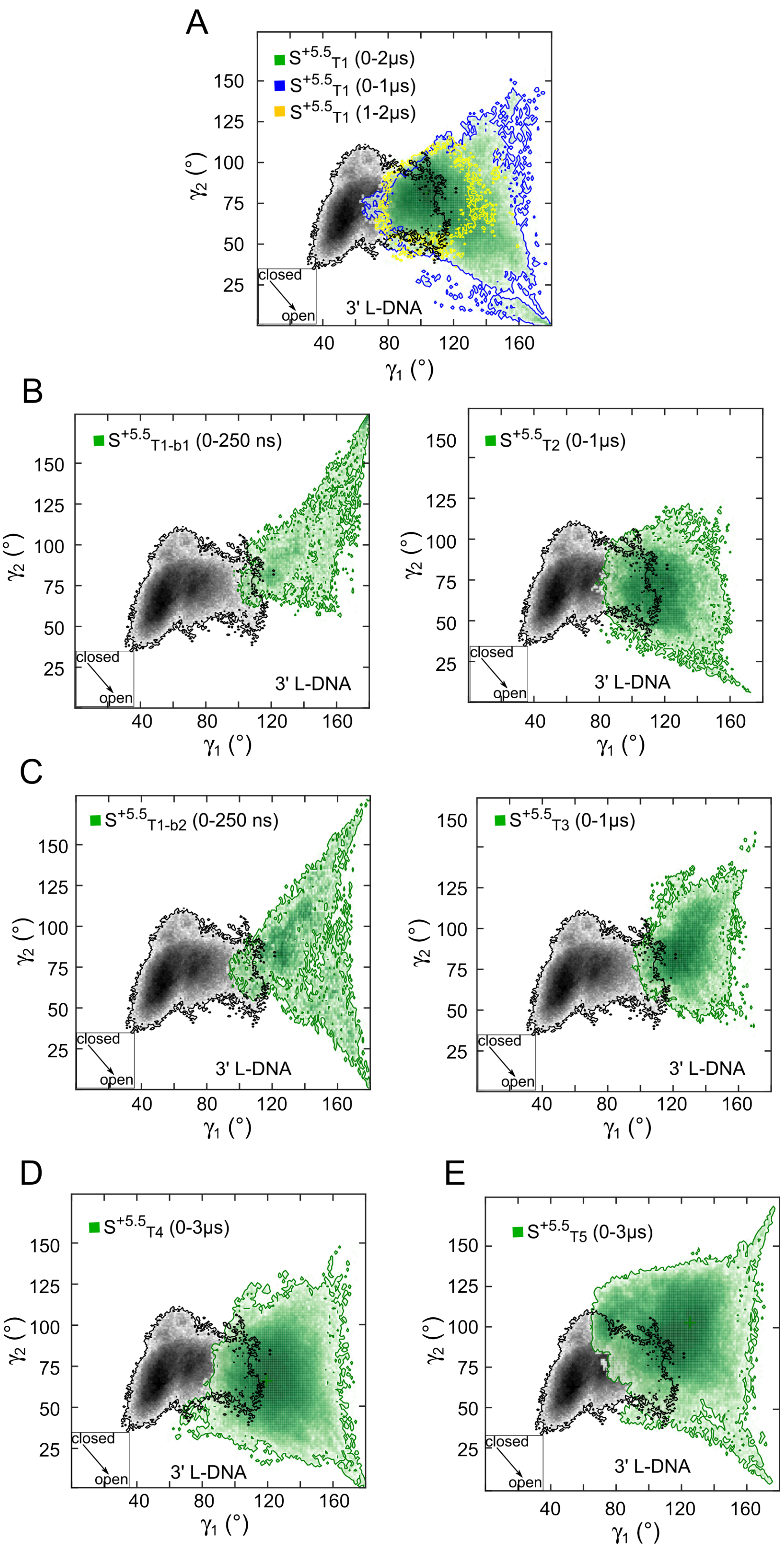

Figure S7: Breathing motions of ESRRB in classical and biased simulations of the Oct4-ESRRB complex started with H3 and H2AC tail configurations selected from the open nucleosome conformation found in $S^{5.5}{}_{1}$. 2D histograms as in Figures 3, S4, and S5 depicting the sampling of the γ_1/γ_2 conformational space from the simulations $S^{5.5}{}_{T1}$ (A), $S^{5.5}{}_{T1\text{-}b1}$, $S^{5.5}{}_{T2}$ (B), $S^{5.5}{}_{T1\text{-}b2}$, $S^{5.5}{}_{T3}$ (C), $S^{5.5}{}_{T4}$ (D), and $S^{5.5}{}_{T5}$ (E). See also Table 1 and Figure 3.

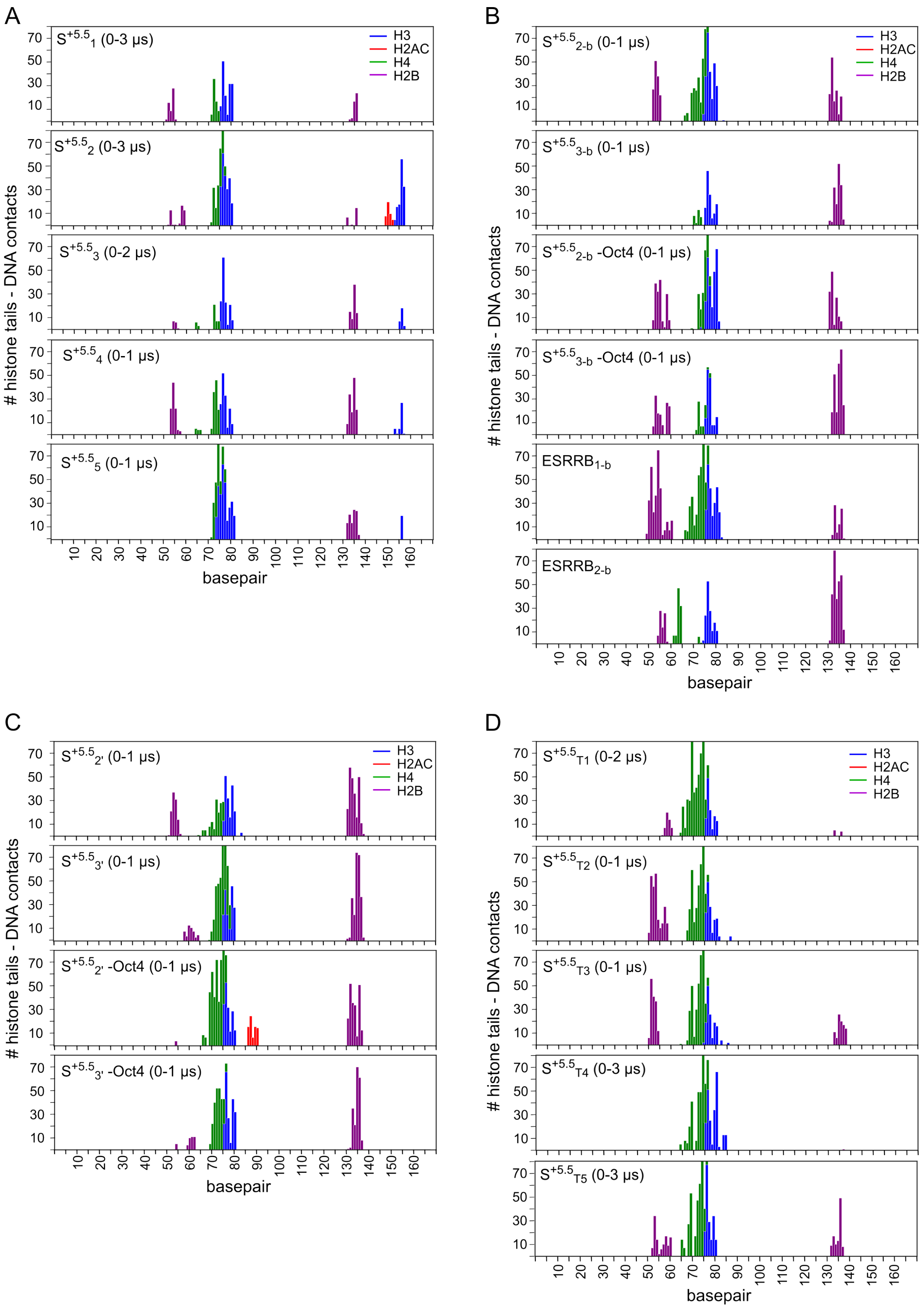

Figure S8: Histone tail - DNA interaction profiles in ESRRB and the Oct4-ESRRB complex. The plots are similar to those in Figure S7. (**A**) The initial 5 classical simulations. (**B**) The biased simulations (**C**) The classical simulations started after the biased simulations from B. (**D**) The classical simulations started with the H3 and H3AC tail configuration selected from the open nucleosome conformation. See also Table 1, S1 and Figures 4, 5.

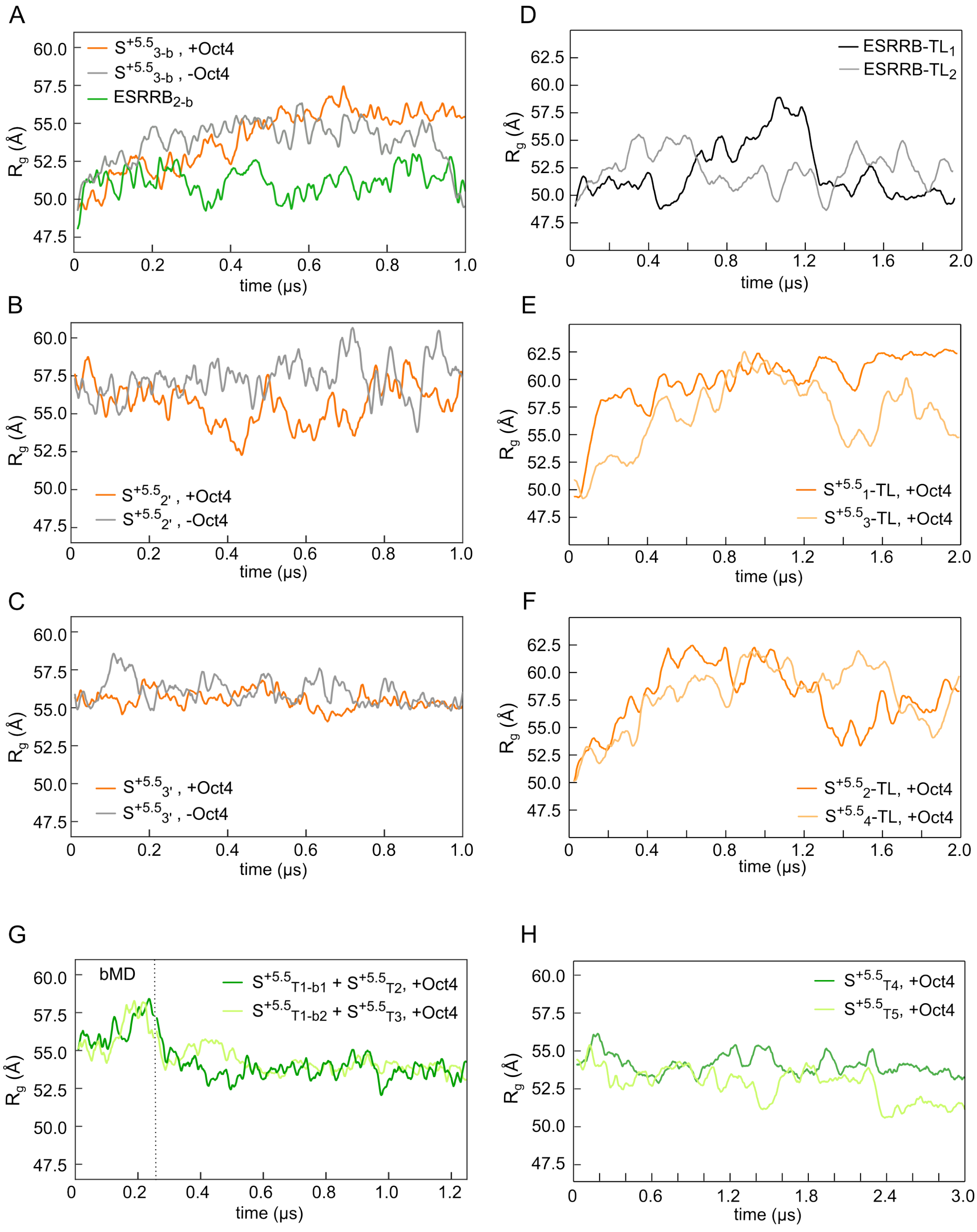

Figure S9: ESRRB nucleosome opening in different simulations. (**A-C**) Biased simulations followed by classical simulations of the Oct4-ESRRB complex. Time series of R_g are calculated from: biased simulations (A) of the ESRRB alone ($ESRRB_{2\text{-b}}$ (green) started from $ESRRB_2$) and of the Oct4-ESRRB started from $S^{5.5}{}_3$ with (orange) and without Oct4 (grey); classical simulations with (orange) and without (grey) Oct4 started from the biased $S^{5.5}{}_{2\text{-b}}$ simulation (B); classical simulations with (orange) and without (grey) Oct4 started from the biased $S^{5.5}{}_{3\text{-b}}$ simulation (C); (**D-F**) Classical simulations with tail-less free (D) and Oct4-bound (E,F) ESRRB nucleosome.(**G-H**) Simulations started with H3 and H2AC tail configurations selected from open an nucleosome conformation. Biased $S^{5.5}{}_{T1\text{-b1}}$ followed by classical $S^{5.5}{}_{T2}$ (dark green) and biased $S^{5.5}{}_{T1\text{-b2}}$ followed by classical $S^{5.5}{}_{T3}$ (light green) (G); additional classical replicates of $S^{5.5}{}_{T1}$ ($S^{5.5}{}_{T4}$ in dark green and $S^{5.5}{}_{T5}$ in light green) (H). See also Table 1, Supplementary Table S1 and Figure 5.

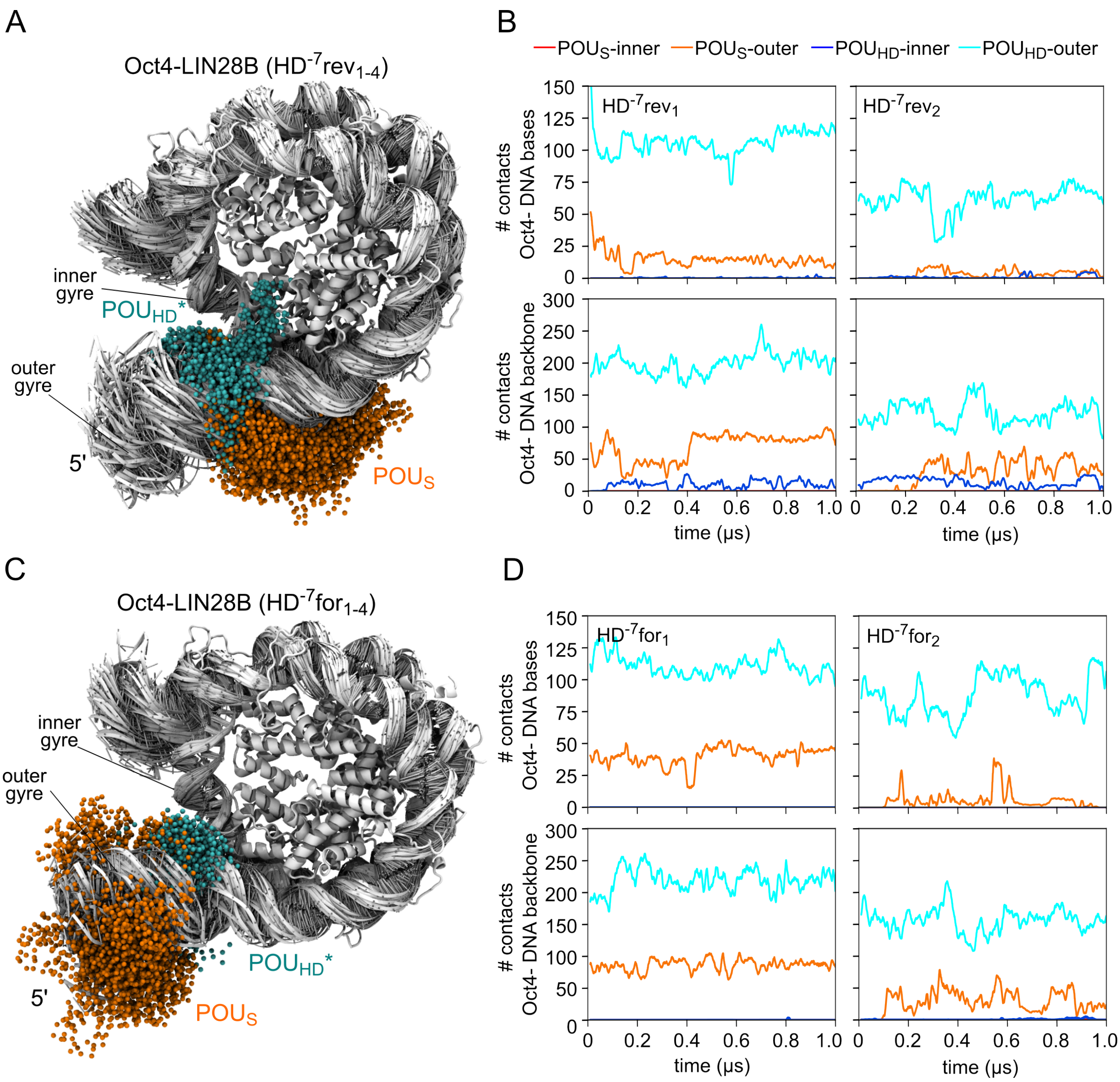

Figure S10: Sequence specific interactions of the POU_{HD} and nonspecific interactions of the POU_S with LIN28B. (**A**) Nucleosome sampling by the Oct4 subdomains in 4 μs aggregate simulation time with Oct4 bound to the HD^{-7} site in reverse orientation. (**B**) Number of contacts between the POU_S (red, orange) or the POU_{HD} (blue, cyan) with DNA bases (sequence specific, upper plots) or DNA backbone (non-specific, lower plots) of the inner gyre (red, blue) or the outer gyre (orange, cyan) in two of the simulations shown in (A) ($HD^{-7}rev_1$ and $HD^{-7}rev_2$). (**A**) Nucleosome sampling by the Oct4 subdomains in 4 μs aggregate simulations time with Oct4 bound to the HD^{-7} site in forward orientation. (**D**) Same as (B) but in two of the simulations shown in (C) ($HD^{-7}for_1$ and $HD^{-7}for_2$). See also Tables 1, S1 and Figure 6.
